# Linear Static Behavior of Damaged Laminated Composite Plates and Shells

**DOI:** 10.3390/ma10070811

**Published:** 2017-07-17

**Authors:** Francesco Tornabene, Nicholas Fantuzzi, Michele Bacciocchi

**Affiliations:** DICAM—Department, School of Engineering and Architecture, University of Bologna, Viale del Risorgimento 2, 40136 Bologna, Italy; nicholas.fantuzzi@unibo.it (N.F.); michele.bacciocchi@unibo.it (M.B.)

**Keywords:** laminated and sandwich structures, damage, stress and strain recovery procedure, generalized differential quadrature method, higher-order shear deformation theories

## Abstract

A mathematical scheme is proposed here to model a damaged mechanical configuration for laminated and sandwich structures. In particular, two kinds of functions defined in the reference domain of plates and shells are introduced to weaken their mechanical properties in terms of engineering constants: a two-dimensional Gaussian function and an ellipse shaped function. By varying the geometric parameters of these distributions, several damaged configurations are analyzed and investigated through a set of parametric studies. The effect of a progressive damage is studied in terms of displacement profiles and through-the-thickness variations of stress, strain, and displacement components. To this end, a posteriori recovery procedure based on the three-dimensional equilibrium equations for shell structures in orthogonal curvilinear coordinates is introduced. The theoretical framework for the two-dimensional shell model is based on a unified formulation able to study and compare several Higher-order Shear Deformation Theories (HSDTs), including Murakami’s function for the so-called zig-zag effect. Thus, various higher-order models are used and compared also to investigate the differences which can arise from the choice of the order of the kinematic expansion. Their ability to deal with several damaged configurations is analyzed as well. The paper can be placed also in the field of numerical analysis, since the solution to the static problem at issue is achieved by means of the Generalized Differential Quadrature (GDQ) method, whose accuracy and stability are proven by a set of convergence analyses and by the comparison with the results obtained through a commercial finite element software.

## 1. Introduction

Shell structures are becoming very popular due to their typical curved shapes that can characterize the structural behavior of these elements. For instance, the capability of transferring and holding external loads is substantially different from the one of a flat panel. Analogously, the free vibrations, stresses and strains, as well as the buckling loads, are highly affected by the shell curvature [1,2]. It should be pointed out that the geometry of a shell structure is completely defined when the corresponding middle surface is accurately described [1,2,3]. The characterization of these shapes is often linked to the greatest issues that could be encountered during the mechanical analysis of shells. These difficulties are even bigger if a doubly-curved surface must be studied, since each point of the domain is defined by two different radii of curvature. To the best of the authors’ knowledge, this obstacle can be overcome if the doubly-curved surface at issue is described analytically by means of the differential geometry principles, as illustrated in the book by Kraus [3].

The mechanical behavior of these structures is even more intriguing to analyze if composite materials are introduced [1,4]. Indeed, the structural response of a composite shell is completely different from the corresponding structure made of conventional materials, such as isotropic mediums. The aim of these innovative materials is to enhance the mechanical behavior of the structure by combining two or more constituents, which could not provide the same response if taken separately. That is the case of a fiber-reinforced material. In this example, high-strength fibers are combined with an isotropic matrix. In general, the fibers (such as carbon or glass filaments) carry the external stresses, whereas the matrix keeps them together and acts as a shield to protect the reinforcing phase from the environmental factors, which could cause a deterioration of mechanical properties [4]. A stack of these fiber-reinforced mediums can be made to obtain the so-called laminated composites. In this circumstance, each layer (or ply) represents one of the various constituent of the composite. As a consequence, the structural response is affected by the number of layers, the orientation of the fibers, and the way they are combined. In general, each fiber-reinforced lamina is orthotropic, thus the corresponding constitutive elastic equations are required to describe properly the relation between stresses and strains. The mechanical analysis of laminated composite plates and shells is currently a recurring topic in the pertinent literature. In particular, the effect of the fiber orientation and the stacking sequence on the structural response has been hugely investigated in many papers to analyze the static [5,6,7,8,9,10,11,12,13,14,15,16,17] and dynamic [18,19,20,21,22,23,24,25,26,27,28,29,30,31,32,33,34,35,36,37,38,39,40,41,42,43] behavior of such structures.

In recent years, the idea of variable mechanical properties for a composite medium has begun to spread in order to optimize the structural performances and reach the best mechanical responses towards the environmental demands. Firstly, the well-known class of functionally graded materials (FGMs) should be mentioned [44,45,46,47,48,49,50,51,52,53,54,55,56,57,58,59,60], where a continuous variation of the material properties along the thickness of the structure is introduced to reduce the stress peaks at the layer interfaces of a laminate. Indeed, this gradual variation of the mechanical properties is applied to overcome some of the limitations of laminated composites, such as delamination and interlaminar cracks. Starting from the same ideas of FGMs, the class of functionally-graded Carbon Nanotube-reinforced composites is becoming very popular due to the introduction of nanoparticles as the reinforcing phase in the composite medium [61,62,63,64,65,66,67,68,69]. Even in this circumstance, the variation of the mechanical properties is obtained by defining the volume fraction distribution of the constituents by means of different through-the-thickness laws. It is clear that the mechanical properties of such composites turn out to be functions of the thickness coordinate. Alternatively, a different kind of composite material has been developed to obtain a variation of its properties within the domain defined by the middle surface of the structure. Such variability can be achieved by changing the orientation of the fiber in each point of the domain. This approach is known in the literature as variable angle tow (VAT) concept [70,71,72,73,74,75,76,77,78,79,80,81,82,83]. Similar variations could be obtained by varying the spatial distribution of the reinforcing fibers by increasing (or decreasing) the space among them [84], or adding a fiber-reinforced ply only in some areas of the domain [85]. This last example can be generalized by applying a smooth thickness profile to the composite structure at issue. As illustrated in [86,87,88,89,90,91,92,93,94], the thickness variation can affect the structural response through an optimal distribution of the materials.

The present paper also falls within the topic of variable stiffness structures, since it aims to investigate the static behavior of composite plates and shells with variable mechanical properties. Nevertheless, this variation is concentrated in some delimited areas of the composite to model a sudden deterioration of the material properties. The weakening at issue can occur when damage spreads within the medium that composes the structure. Consequently, the elastic properties of the structure are considerably reduced only in limited zones, whereas the undamaged areas of the structure present unchanged features. This configuration can be mathematically achieved by assigning particular smooth functions, such as the well-known two-dimensional Gaussian distribution or an ellipse shaped law, to the elastic parameters that define the mechanical properties of the structure. As highlighted in the paper by Ladevèze and Le Dantec [95], a generic damage can be described as a reduction of the material stiffness originating from microcracking and debonding, for instance. Different kinds of damage are also possible, as presented by Daudeville and Ladevèze [96], such as transverse matrix cracking and fiber ruptures. A complete review of the failures that can occur in a laminated composite medium is presented in the books by Reddy and Miravete [97] and by Murakami [98], in which it is specified also that the main issues in developing a mathematical model for damage are related to the various geometric scales involved in the failure progression. Independently from the scale of the damage, a failure is always associated with a global stiffness reduction of the structure. The stiffness changes at issue affect deflections, vibration characteristics, and the stress and strains distribution, as illustrated clearly in the work by Highsmith and Reifsnider [99]. In all the mentioned works, the reasons that cause damage are diverse. Since many variables are involved in the growth and progression of damage, this topic is often investigated from both the numerical [100,101,102,103,104,105,106,107] and experimental [108,109,110,111,112,113] points of view. Analogously, several mechanical approaches at a different scale were also proposed. Typically, the most exploited ones are variational methods [97], continuum damage models [97,114,115,116,117], or approaches that take into account a plastic behavior of the structure [95,97,118,119]. The more appropriate approach should be chosen according to the failure to investigate. It should be mentioned that the causes that give rise to a damage have not been investigated in the present paper. Analogously, the point of the domain in which this damage arises is assumed a priori. Bearing in mind these hypotheses, several investigations are presented in this research to show the effect of the damage parameters (point of application, intensity, and width) on the linear static behavior of laminated shell structures.

Once the constitutive laws are specified, the governing equations are obtained in the framework of higher-order shear deformation theories (HSDTs), since these peculiar mechanical configurations could be ineffectively studied through the well-known first-order shear deformation theory (FSDT), as highlighted in many works during the last decades [61,78,79,80,92,93,94,120,121,122,123,124,125,126]. Thus, an analytical model able to deal with several enriched displacement fields is employed to describe the mechanical behavior of these structures. The fundamental assumptions of this theoretical formulation can be found in [1,5,18,19,20,127,128,129]. As far as the achievement of the solutions is concerned, a computational method is introduced. In particular, the strong form of the governing equation is solved numerically by means of the Generalized Differential Quadrature (GDQ) method [130,131]. The same numerical scheme is employed to evaluate both the geometric parameters of the doubly-curved surfaces used as reference domains and the through-the-thickness variations of stress, strain, and displacement components. For this purpose, a posteriori recovery procedure based on the three-dimensional equilibrium equations is proposed.

## 2. Shell Geometry

**Figure 1:**
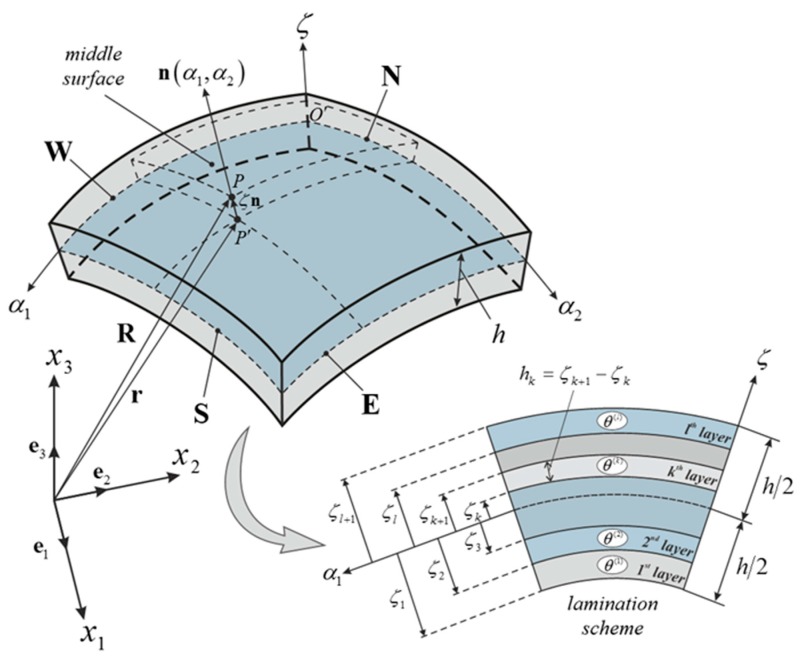
Doubly-curved shell element, edge identification and lamination scheme. The notation $\left(\theta^{\left(1\right)}/\theta^{\left(2\right)}/\ldots /\theta^{\left(k\right)}/\ldots /\theta^{\left(l\right)}\right)$ represents the stacking sequence of the composite, where $\theta^{\left(k\right)}$ is the orientation of the k -th layer.

In this paper, differential geometry is used to evaluate those geometric parameters needed for the description of the shell middle surface, which are required in the fundamental operators of the governing equations [1,3]. Let us consider a generic shell element in a global reference system $O\;x_{1}\;x_{2}\;x_{3}$ as shown in Figure 1. Each point P within the three-dimensional medium of thickness h is identified by the vector $R\left(\alpha_{1},\alpha_{2},\zeta \right)$, which takes the following aspect

(1) $$R\left(\alpha_{1},\alpha_{2},\zeta \right)=r\left(\alpha_{1},\alpha_{2}\right)+\zeta \;n\left(\alpha_{1},\alpha_{2}\right)$$

The coordinate ζ specifies the normal direction along the shell thickness, whereas $\alpha_{1},\;\alpha_{2}$ are the orthogonal and principal curvilinear coordinates of the shell middle surface. By hypothesis, the coordinates $\alpha_{1},\;\alpha_{2}$ coincide with the parametric lines of curvature of the middle surface. It should be specified that their meaning is different according to the surface to describe [1,92,93,94]. As shown in Figure 1, $O\;\alpha_{1}\;\alpha_{2}\;\zeta$ denotes the local reference system of the reference surface of the shell, which corresponds to its middle surface.

Each point *P*’ of the shell middle surface is located by the position vector $r\left(\alpha_{1},\alpha_{2}\right)$. Once this position vector is defined, its derivatives with respect to $\alpha_{1},\;\alpha_{2}$ are required. For the first two orders, one gets

(2) $$r_{,1}=\frac{\partial r}{\partial \alpha_{1}},\;r_{,2}=\frac{\partial r}{\partial \alpha_{2}},\;r_{,11}=\frac{\partial^{2}r}{\partial \alpha_{1}^{2}},\;r_{,22}=\frac{\partial^{2}r}{\partial \alpha_{2}^{2}}$$

The normal direction ζ is identified by the following outward unit normal vector $n\left(\alpha_{1},\alpha_{2}\right)$, described by the following vector product

(3) $$n=\frac{r_{,1}\times r_{,2}}{\left|r_{,1}\times r_{,2}\right|}$$

Once the position vector $r\left(\alpha_{1},\alpha_{2}\right)$ is provided, by means of quantities in Equation (2), the well-known Lamè parameters $A_{\;1}\left(\alpha_{1},\alpha_{2}\right)$, $A_{\;2}\left(\alpha_{1},\alpha_{2}\right)$ are computed as scalar products

(4) $$A_{\;1}=\sqrt{r_{,1}\cdot r_{,1}},\;A_{\;2}=\sqrt{r_{,2}\cdot r_{,2}}$$

Definitions (4) are based on differential geometry principles. It should be noted that the scalar parameters in (4) are clearly physical quantities whose magnitude depends on the coordinates $\alpha_{1},\alpha_{2}$ defined on the reference domain (middle surface). Further details concerning the Lamè parameters are illustrated in the book by Tornabene et al. [1].

For a doubly-curved surface, the principal radii of curvature $R_{\;1}\left(\alpha_{1},\alpha_{2}\right)$, $R_{\;2}\left(\alpha_{1},\alpha_{2}\right)$ are also required. The definitions below are valid if the coordinates $\alpha_{1},\;\alpha_{2}$ are orthogonal and principal

(5) $$R_{\;1}=-\frac{r_{,1}\cdot r_{,1}}{r_{,11}\cdot n},\;R_{\;2}=-\frac{r_{,2}\cdot r_{,2}}{r_{,22}\cdot n}$$

In case of a singly-curved shell or a degenerate shell (which corresponds to a plate), one radius of curvature (or both of them for a flat structure) tends to infinite value. Finally, the geometric quantities $H_{1}\left(\alpha_{1},\alpha_{2},\zeta \right)$, $H_{2}\left(\alpha_{1},\alpha_{2},\zeta \right)$ are also needed. They are defined as follows

(6) $$H_{1}=1+\frac{\zeta }{R_{\;1}},\;H_{2}=1+\frac{\zeta }{R_{\;2}}$$

and they are introduced to take into account the three-dimensional size effect related to the shell curvature. It should be stated that a finite domain is specified by setting limited values along each principal coordinate. In particular, the limitations at issue can be expressed analytically as follows

(7) $$\alpha_{1}^{0}\leq \alpha_{1}\leq \alpha_{1}^{1},\;\alpha_{2}^{0}\leq \alpha_{2}\leq \alpha_{2}^{1},\;-\frac{h}{2}\leq \zeta \leq \frac{h}{2}$$

in which $\alpha_{i}^{0},\alpha_{i}^{1}$, for i=1,2, represent the minimum and the maximum boundary values along the coordinates $\alpha_{1},\;\alpha_{2}$, respectively. When a laminated composite shell is considered, the overall thickness of the structure is given by

(8) $$h=\sum_{k=1}^{l}h_{k}$$

where l is the total number of layers, whereas the index k stands for the geometric and mechanical parameters of the k-th ply. As it can be noted from Figure 1, $h_{k}$ represents the thickness of the k-th ply and it can be evaluated as follows

(9) $$h_{k}=\zeta_{k+1}-\zeta_{k}$$

in which $\zeta_{k}$ and $\zeta_{k+1}$ are the lower and upper coordinates of the k-th layer, respectively.

## 3. Shell Structural Model

The displacement field of a generic laminated composite shell is defined by the three-dimensional displacements $U_{1}\left(\alpha_{1},\alpha_{2},\zeta \right)$, $U_{2}\left(\alpha_{1},\alpha_{2},\zeta \right)$, $U_{3}\left(\alpha_{1},\alpha_{2},\zeta \right)$, which assume the following aspect

(10) $$\begin{array}{l} U_{1}=\sum_{\tau =0}^{N+1}F_{\tau }^{}u_{1}^{\left(\tau \right)}\\ U_{2}=\sum_{\tau =0}^{N+1}F_{\tau }^{}u_{2}^{\left(\tau \right)}\\ U_{3}=\sum_{\tau =0}^{N+1}F_{\tau }^{}u_{3}^{\left(\tau \right)} \end{array}$$

in which the order of kinematic expansion τ can be chosen arbitrarily. For conciseness purposes, the displacement components in Equation (10) can be collected into the vector $U=U\left(\alpha_{1},\alpha_{2},\zeta \right)$. According to the present formulation, which is able to deal with both the shear deformations and the stretching effect along the thickness of the structure, several HSDTs can be obtained by setting the maximum order of expansion N. The degrees of freedom of the model are given by the generalized displacements of the shell middle surface $u_{1}^{\left(\tau \right)}\left(\alpha_{1},\alpha_{2}\right)$, $u_{2}^{\left(\tau \right)}\left(\alpha_{1},\alpha_{2}\right)$, $u_{3}^{\left(\tau \right)}\left(\alpha_{1},\alpha_{2}\right)$, which can be collected in the corresponding vector $u^{\left(\tau \right)}\left(\alpha_{1},\alpha_{2}\right)$

(11) $$u^{\left(\tau \right)}={\left[\begin{array}{ccc} u_{1}^{\left(\tau \right)} & u_{2}^{\left(\tau \right)} & u_{3}^{\left(\tau \right)} \end{array}\right]}^{T}$$

for τ=0,1,2,…,N,N+1. It should be specified that the present theory belongs to the class of Equivalent Single Layer (ESL) approaches, since all the geometric and mechanical parameters are evaluated on the middle surface of the structure. It is also important to specify that only the orders τ=0,1 have a physical meaning. In particular, the translational displacement along $\alpha_{1},\alpha_{2},\zeta$ are obtained for τ=0, whereas τ=1 provides the corresponding rotational components. Further details about these aspects can be found in the book by Tornabene et al. [1]. The kinematic model in (10) is well-defined once the shear functions (or thickness functions) $F_{\tau }\left(\zeta \right)$ are specified for each order of kinematic expansion. A complete list of functions that can be chosen for this purpose is shown in [5]. In this paper, the power-law function $\zeta^{\;\tau }$ is used to define the displacement field up to the N-th order of expansion. As far as the (N+1)-th expansion order is concerned, the corresponding thickness function $F_{N+1}\left(\zeta \right)$ coincides with the well-known Murakami’s function Z=Z(ζ), whose analytical expression is given by

(12) $$Z={\left(-1\right)}^{k}z_{k}$$

where the dimensionless parameter $z_{k}\left(\zeta \right)\in \left[-1,1\right]$ is defined as

(13) $$z_{k}=\left(\frac{2}{\zeta_{k+1}-\zeta_{k}}\zeta -\frac{\zeta_{k+1}+\zeta_{k}}{\zeta_{k+1}-\zeta_{k}}\right)$$

This function could be required to capture the so-called zig-zag effect that could happen when peculiar lamination schemes are considered. Further details concerning the Murakami’s function can be found in [92,93,94,129,130]. For the sake of completeness, it should be mentioned that this function allows the description of continuous three-dimensional displacements characterized by discontinuities in their derivatives at the interface between two adjacent layers. To sum up, the thickness functions are chosen as follows, for the corresponding orders of kinematic expansion

(14) $$F_{\tau }\left(\zeta \right)=\left\{ \begin{array}{ll} \zeta^{\tau } & \mathrm{for}\;\tau =0,1,\ldots ,N\\ {\left(-1\right)}^{k}z_{k} & \mathrm{for}\;\tau =N+1 \end{array}\right.$$

It is clear that the maximum order of expansion *N* defines the structural model. Consequently, the acronyms ED N and EDZ N are used to specify the theory. As illustrated in the previous papers [93,94,95], the letter “E” specifies that the theory is based on an ESL approach, whereas the letter “D” states that the governing equations are deduced in terms of the generalized displacements. The letter “Z” is added to the notation only when the Murakami’s function is embedded in the model.

The generalized strains of the middle surface $\varepsilon^{\left(\tau \right)}\left(\alpha_{1},\alpha_{2}\right)$ are evaluated for each order τ as a function of the generalized displacements as follows

(15) $$\varepsilon^{\left(\tau \right)}=D_{\Omega }^{}u^{\left(\tau \right)}$$

in which the kinematic operator $D_{\Omega }$ is given by

(16) $$D_{\Omega }={\left[\begin{array}{ccccccccc} \frac{1}{A_{\;1}}\frac{\partial }{\partial \alpha_{1}} & \frac{1}{A_{\;1}A_{\;2}}\frac{\partial A_{\;2}}{\partial \alpha_{1}} & -\frac{1}{A_{\;1}A_{2}}\frac{\partial A_{1}}{\partial \alpha_{2}} & \frac{1}{A_{\;2}}\frac{\partial }{\partial \alpha_{2}} & -\frac{1}{R_{\;1}} & 0 & 1 & 0 & 0\\ \frac{1}{A_{\;1}A_{\;2}}\frac{\partial A_{\;1}}{\partial \alpha_{2}} & \frac{1}{A_{\;2}}\frac{\partial }{\partial \alpha_{2}} & \frac{1}{A_{\;1}}\frac{\partial }{\partial \alpha_{1}} & -\frac{1}{A_{\;1}A_{2}}\frac{\partial A_{2}}{\partial \alpha_{1}} & 0 & -\frac{1}{R_{\;2}} & 0 & 1 & 0\\ \frac{1}{R_{\;1}} & \frac{1}{R_{\;2}} & 0 & 0 & \frac{1}{A_{\;1}}\frac{\partial }{\partial \alpha_{1}} & \frac{1}{A_{\;2}}\frac{\partial }{\partial \alpha_{2}} & 0 & 0 & 1 \end{array}\right]}^{T}$$

For the sake of completeness, the quantities collected in the generalized strain vector are shown below

(17) $$\varepsilon^{\left(\tau \right)}={\left[\begin{array}{ccccccccc} \varepsilon_{1}^{\left(\tau \right)} & \varepsilon_{2}^{\left(\tau \right)} & \gamma_{1}^{\left(\tau \right)} & \gamma_{2}^{\left(\tau \right)} & \gamma_{13}^{\left(\tau \right)} & \gamma_{23}^{\left(\tau \right)} & \omega_{13}^{\left(\tau \right)} & \omega_{23}^{\left(\tau \right)} & \varepsilon_{3}^{\left(\tau \right)} \end{array}\right]}^{T}$$

The complete treatise concerning the generalized strains and their definitions in extended notation can be found in the book by Tornabene et al. [1]. The meaning of higher-order terms is also explained. Quantities in (17) allow the evaluation also of the three-dimensional strains of the structure $\varepsilon \left(\alpha_{1},\alpha_{2},\zeta \right)$

(18) $$\varepsilon =\sum_{\tau =0}^{N+1}Z^{\left(\tau \right)}\varepsilon^{\left(\tau \right)}$$

where the strain vector is given by

(19) $$\varepsilon ={\left[\begin{array}{cccccc} \varepsilon_{1} & \varepsilon_{2} & \gamma_{12} & \gamma_{1n} & \gamma_{2n} & \varepsilon_{n} \end{array}\right]}^{T}$$

whereas the matrix $Z^{\left(\tau \right)}\left(\zeta \right)$ assumes the following form

(20) $$Z^{\left(\tau \right)}=\left[\begin{array}{ccccccccc} \frac{F_{\tau }^{}}{H_{1}} & 0 & 0 & 0 & 0 & 0 & 0 & 0 & 0\\ 0 & \frac{F_{\tau }^{}}{H_{2}} & 0 & 0 & 0 & 0 & 0 & 0 & 0\\ 0 & 0 & \frac{F_{\tau }^{}}{H_{1}} & \frac{F_{\tau }^{}}{H_{2}} & 0 & 0 & 0 & 0 & 0\\ 0 & 0 & 0 & 0 & \frac{F_{\tau }^{}}{H_{1}} & 0 & \frac{\partial F_{\tau }^{}}{\partial \zeta } & 0 & 0\\ 0 & 0 & 0 & 0 & 0 & \frac{F_{\tau }^{}}{H_{2}} & 0 & \frac{\partial F_{\tau }^{}}{\partial \zeta } & 0\\ 0 & 0 & 0 & 0 & 0 & 0 & 0 & 0 & \frac{\partial F_{\tau }^{}}{\partial \zeta } \end{array}\right]$$

As far as the linear elastic constitutive relations are concerned, the stress components $\sigma^{\left(k\right)}\left(\alpha_{1},\alpha_{2},\zeta \right)$ for the generic k-th orthotropic layer can be written as follows

(21) $$\sigma^{\left(k\right)}={\bar{C}}^{\left(k\right)}\varepsilon^{\left(k\right)}$$

where the stress vector is given by

(22) $$\sigma^{\left(k\right)}={\left[\begin{array}{cccccc} \sigma_{1}^{\left(k\right)} & \sigma_{2}^{\left(k\right)} & \tau_{12}^{\left(k\right)} & \tau_{1n}^{\left(k\right)} & \tau_{2n}^{\left(k\right)} & \sigma_{n}^{\left(k\right)} \end{array}\right]}^{T}$$

whereas the matrix ${\bar{C}}^{\left(k\right)}\left(\alpha_{1},\alpha_{2}\right)$ collects the elastic coefficients as shown below

(23) $${\bar{C}}^{\left(k\right)}=\left[\begin{array}{cccccc} {\bar{E}}_{11}^{\left(k\right)} & {\bar{E}}_{12}^{\left(k\right)} & {\bar{E}}_{16}^{\left(k\right)} & 0 & 0 & {\bar{E}}_{13}^{\left(k\right)}\\ {\bar{E}}_{12}^{\left(k\right)} & {\bar{E}}_{22}^{\left(k\right)} & {\bar{E}}_{26}^{\left(k\right)} & 0 & 0 & {\bar{E}}_{23}^{\left(k\right)}\\ {\bar{E}}_{16}^{\left(k\right)} & {\bar{E}}_{26}^{\left(k\right)} & {\bar{E}}_{66}^{\left(k\right)} & 0 & 0 & {\bar{E}}_{36}^{\left(k\right)}\\ 0 & 0 & 0 & {\bar{E}}_{44}^{\left(k\right)} & {\bar{E}}_{45}^{\left(k\right)} & 0\\ 0 & 0 & 0 & {\bar{E}}_{45}^{\left(k\right)} & {\bar{E}}_{55}^{\left(k\right)} & 0\\ {\bar{E}}_{13}^{\left(k\right)} & {\bar{E}}_{23}^{\left(k\right)} & {\bar{E}}_{36}^{\left(k\right)} & 0 & 0 & {\bar{E}}_{33}^{\left(k\right)} \end{array}\right]$$

The elements of the constitutive matrix ${\bar{E}}_{nm}^{\left(k\right)}\left(\alpha_{1},\alpha_{2}\right)$ represent the material constants. They can be assumed equal to the reduced elastic coefficients if the hypothesis of plane stress is introduced

(24) $${\bar{E}}_{nm}^{\left(k\right)}={\bar{Q}}_{nm}^{\left(k\right)}$$

Conversely, they are set equal to the non-reduced coefficients

(25) $${\bar{E}}_{nm}^{\left(k\right)}={\bar{C}}_{nm}^{\left(k\right)}$$

It should be noted that the normal stress $\sigma_{n}^{}$ and the normal strain $\varepsilon_{n}$ are neglected if the plane stress hypothesis is introduced. Independently from this assumption, the elastic coefficients ${\bar{E}}_{nm}^{\left(k\right)}$ must be evaluated in the geometric local reference system $O\;\alpha_{1}\;\alpha_{2}\;\zeta$. This aspect is extremely important when each orthotropic layer of the laminate has a different orientation. By means of the proper relations that allow the orientation in hand to be taken into account [1,4], the elastic coefficients can be evaluated as a function of the corresponding elastic coefficients related to the local reference system of the oriented ply $C_{nm}^{\left(k\right)}$ (or $Q_{nm}^{\left(k\right)}$ for the reduced coefficients). These quantities are given in terms of the engineering constants of the medium, which are the Young’s moduli ($E_{1}^{\left(k\right)}$, $E_{2}^{\left(k\right)}$, $E_{3}^{\left(k\right)}$), the shear moduli ($G_{12}^{\left(k\right)}$, $G_{13}^{\left(k\right)}$, $G_{23}^{\left(k\right)}$), and the Poisson’s ratios ($\nu_{12}^{\left(k\right)}$, $\nu_{13}^{\left(k\right)}$, $\nu_{23}^{\left(k\right)}$). The plane stress-reduced elastic coefficients written in the material reference system are defined as follows

(26) $$\begin{array}{c} Q_{11}^{\left(k\right)}=\frac{E_{1}^{\left(k\right)}}{1-\nu_{12}^{\left(k\right)}\nu_{21}^{\left(k\right)}},\;Q_{22}^{\left(k\right)}=\frac{E_{2}^{\left(k\right)}}{1-\nu_{12}^{\left(k\right)}\nu_{21}^{\left(k\right)}},\;Q_{12}^{\left(k\right)}=\frac{\nu_{12}^{\left(k\right)}E_{2}^{\left(k\right)}}{1-\nu_{12}^{\left(k\right)}\nu_{21}^{\left(k\right)}}\\ Q_{66}^{\left(k\right)}=G_{12}^{\left(k\right)},\;Q_{44}^{\left(k\right)}=G_{13}^{\left(k\right)},\;Q_{55}^{\left(k\right)}=G_{23}^{\left(k\right)} \end{array}$$

The following expression must be introduced instead for the non-reduced coefficients

(27) $$\begin{array}{c} C_{11}^{\left(k\right)}=\frac{1-\nu_{23}^{\left(k\right)}\nu_{32}^{\left(k\right)}}{E_{2}^{\left(k\right)}E_{3}^{\left(k\right)}\Delta_{}^{\left(k\right)}},\;C_{12}^{\left(k\right)}=\frac{\nu_{21}^{\left(k\right)}+\nu_{31}^{\left(k\right)}\nu_{23}^{\left(k\right)}}{E_{2}^{\left(k\right)}E_{3}^{\left(k\right)}\Delta_{}^{\left(k\right)}},\;C_{13}^{\left(k\right)}=\frac{\nu_{31}^{\left(k\right)}+\nu_{21}^{\left(k\right)}\nu_{32}^{\left(k\right)}}{E_{2}^{\left(k\right)}E_{3}^{\left(k\right)}\Delta_{}^{\left(k\right)}}\\ C_{22}^{\left(k\right)}=\frac{1-\nu_{13}^{\left(k\right)}\nu_{31}^{\left(k\right)}}{E_{1}^{\left(k\right)}E_{3}^{\left(k\right)}\Delta_{}^{\left(k\right)}},\;C_{23}^{\left(k\right)}=\frac{\nu_{32}^{\left(k\right)}+\nu_{12}^{\left(k\right)}\nu_{31}^{\left(k\right)}}{E_{1}^{\left(k\right)}E_{3}^{\left(k\right)}\Delta_{}^{\left(k\right)}},\;C_{33}^{\left(k\right)}=\frac{1-\nu_{12}^{\left(k\right)}\nu_{21}^{\left(k\right)}}{E_{1}^{\left(k\right)}E_{2}^{\left(k\right)}\Delta_{}^{\left(k\right)}}\\ C_{44}^{\left(k\right)}=G_{13}^{\left(k\right)},\;C_{55}^{\left(k\right)}=G_{23}^{\left(k\right)},\;C_{66}^{\left(k\right)}=G_{12}^{\left(k\right)} \end{array}$$

in which the quantity $\Delta_{}^{\left(k\right)}$ is given by

(28) $$\Delta_{}^{\left(k\right)}=\frac{1-\nu_{12}^{\left(k\right)}\nu_{21}^{\left(k\right)}-\nu_{23}^{\left(k\right)}\nu_{32}^{\left(k\right)}-\nu_{31}^{\left(k\right)}\nu_{13}^{\left(k\right)}-2\nu_{21}^{\left(k\right)}\nu_{32}^{\left(k\right)}\nu_{13}^{\left(k\right)}}{E_{1}^{\left(k\right)}E_{2}^{\left(k\right)}E_{3}^{\left(k\right)}}$$

With reference to definitions (26)–(28), all the remaining engineering constants can be deduced by means of the following relations

(29) $$\frac{\nu_{ij}^{\left(k\right)}}{E_{i}^{\left(k\right)}}=\frac{\nu_{ji}^{\left(k\right)}}{E_{j}^{\left(k\right)}},\;G_{ij}^{\left(k\right)}=G_{ji}^{\left(k\right)}\;\mathrm{for}\;i,j=1,2,3$$

In addition, the orientation of the material properties $\theta^{\left(k\right)}$ must be specified for each layer for a complete mechanical characterization of the laminate. The notation $\left(\theta^{\left(1\right)}/\theta^{\left(2\right)}/\ldots /\theta^{\left(k\right)}/\ldots /\theta^{\left(l\right)}\right)$ is used to specify the stacking sequence of the composite structure (Figure 1). For completeness purposes, it should be recalled that an isotropic medium requires only two independent engineering constants and its properties are evaluated independently from the orientation of the material reference system. In the present paper, the aspect of damaged structures is introduced. A generic damage can be seen as a relatively concentrated deterioration of the mechanical properties of the elastic medium. Thus, peculiar functions can be introduced to model this rapid variation of the mechanical features of a layer. In particular, each engineering constant is affected by this sudden decay. Analytically speaking, all the engineering constants of the medium are multiplied by the factor $\Psi_{}^{\left(k\right)}$, which assumes the aspect below

(30) $$\Psi_{}^{\left(k\right)}=\left\{ \begin{array}{l} 1-\delta^{\left(k\right)}\exp\left(\psi_{G}^{\left(k\right)}\right)\\ 1-\delta^{\left(k\right)}\exp\left(\psi_{E}^{\left(k\right)}\right) \end{array}\right.$$

where $\delta^{\left(k\right)}\in \left[0,1\right]$ denotes the intensity of the damage. On the other hand, the functions $\psi_{G}^{\left(k\right)}\left(\alpha_{1},\alpha_{2}\right)$, $\psi_{E}^{\left(k\right)}\left(\alpha_{1},\alpha_{2}\right)$ represent the two-dimensional normalized Gaussian function and an elliptic variation, respectively. The variation at issue are defined as follows

(31) $$\psi_{G}^{\left(k\right)}=-\frac{1}{2\left(1-{\left(\rho_{12}^{\left(k\right)}\right)}^{2}\right)}\left({\left(\frac{\alpha_{1}-\alpha_{1m}^{\left(k\right)}}{\Lambda_{1}^{\left(k\right)}}\right)}^{2}+{\left(\frac{\alpha_{2}-\alpha_{2m}^{\left(k\right)}}{\Lambda_{2}^{\left(k\right)}}\right)}^{2}-2\rho_{12}^{\left(k\right)}\frac{\alpha_{1}-\alpha_{1m}^{\left(k\right)}}{\Lambda_{1}^{\left(k\right)}}\frac{\alpha_{2}-\alpha_{2m}^{\left(k\right)}}{\Lambda_{2}^{\left(k\right)}}\right)$$

(32) $$\psi_{E}^{\left(k\right)}=-\left({\left(\frac{\left(\alpha_{1}-\alpha_{1m}^{\left(k\right)}\right)\cos\beta^{\left(k\right)}+\left(\alpha_{2}-\alpha_{2m}^{\left(k\right)}\right)\sin\beta^{\left(k\right)}}{\Lambda_{1}^{\left(k\right)}}\right)}^{2}+{\left(\frac{-\left(\alpha_{1}-\alpha_{1m}^{\left(k\right)}\right)\sin\beta^{\left(k\right)}+\left(\alpha_{2}-\alpha_{2m}^{\left(k\right)}\right)\cos\beta^{\left(k\right)}}{\Lambda_{2}^{\left(k\right)}}\right)}^{2}\right)$$

**Figure 2:**
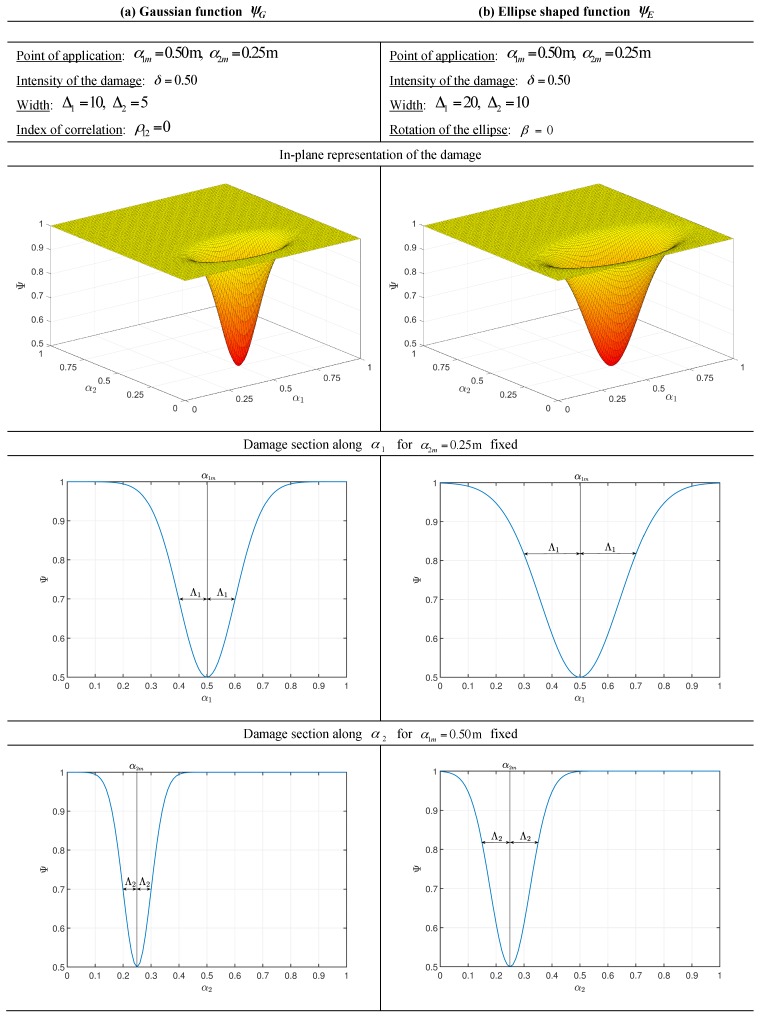
Geometric meaning of the corresponding parameters for the two-dimensional distributions employed to model a generic damage, within a square reference domain with $\alpha_{1},\alpha_{2}\in \left[0,1\;m\right]$: (**a**) Gaussian function; (**b**) ellipse shaped function.

It should be noted that such variations are applied at the point $\left(\alpha_{1m}^{\left(k\right)},\alpha_{2m}^{\left(k\right)}\right)$ of the domain, with $\alpha_{1m}^{\left(k\right)}\in \left[\alpha_{1}^{0},\alpha_{1}^{1}\right]$, $\alpha_{2m}^{\left(k\right)}\in \left[\alpha_{2}^{0},\alpha_{2}^{1}\right]$. On the other hand, the width of the damaged area is controlled by the size parameters $\Lambda_{1}^{\left(k\right)},\Lambda_{2}^{\left(k\right)}$, which can be evaluated as a function of the corresponding quantities $\Delta_{1}^{\left(k\right)},\Delta_{2}^{\left(k\right)}$ as follows

(33) $$\Lambda_{1}^{\left(k\right)}=\frac{\Delta_{1}^{\left(k\right)}}{100}\left(\alpha_{1}^{1}-\alpha_{1}^{0}\right),\;\Lambda_{1}^{\left(k\right)}=\frac{\Delta_{2}^{\left(k\right)}}{100}\left(\alpha_{2}^{1}-\alpha_{2}^{0}\right)$$

with $\Delta_{1}^{\left(k\right)},\Delta_{2}^{\left(k\right)}>0$. Quantities $\rho_{12}^{\left(k\right)}\in \left[-1,1\right]$ and $\beta^{\left(k\right)}\in \left[0,2\pi \right]$ denote respectively the correlation parameter of the Gaussian distribution and the rotation of the ellipse, which is evaluated counterclockwise from the axis $\alpha_{1}$. For the sake of clarity, two examples of the application of the functions at issue are depicted in Figure 2 to understand the meaning of the various geometric quantities just introduced. The superscript k which characterizes each parameter means that the variations can assume different shapes through the various layers of the laminate. As a result, the engineering constants of each layer depend on the local coordinate $\alpha_{1},\alpha_{2}$ of the reference surface as can be observed from the following relations

(34) $$\begin{array}{c} {\bar{E}}_{1}^{\left(k\right)}\left(\alpha_{1},\alpha_{2}\right)=E_{1}^{\left(k\right)}\Psi_{}^{\left(k\right)},\;{\bar{E}}_{2}^{\left(k\right)}\left(\alpha_{1},\alpha_{2}\right)=E_{2}^{\left(k\right)}\Psi_{}^{\left(k\right)},\;{\bar{E}}_{3}^{\left(k\right)}\left(\alpha_{1},\alpha_{2}\right)=E_{3}^{\left(k\right)}\Psi_{}^{\left(k\right)}\\ {\bar{G}}_{12}^{\left(k\right)}\left(\alpha_{1},\alpha_{2}\right)=G_{12}^{\left(k\right)}\Psi_{}^{\left(k\right)},\;{\bar{G}}_{13}^{\left(k\right)}\left(\alpha_{1},\alpha_{2}\right)=G_{13}^{\left(k\right)}\Psi_{}^{\left(k\right)},\;{\bar{G}}_{23}^{\left(k\right)}\left(\alpha_{1},\alpha_{2}\right)=G_{23}^{\left(k\right)}\Psi_{}^{\left(k\right)}\\ {\bar{\nu }}_{12}^{\left(k\right)}\left(\alpha_{1},\alpha_{2}\right)=\nu_{12}^{\left(k\right)}\Psi_{}^{\left(k\right)},\;{\bar{\nu }}_{13}^{\left(k\right)}\left(\alpha_{1},\alpha_{2}\right)=\nu_{13}^{\left(k\right)}\Psi_{}^{\left(k\right)},\;{\bar{\nu }}_{23}^{\left(k\right)}\left(\alpha_{1},\alpha_{2}\right)=\nu_{23}^{\left(k\right)}\Psi_{}^{\left(k\right)} \end{array}$$

Once the generalized strain components (15) are evaluated, it is possible to compute also the stress resultants $S^{\left(\tau \right)}\left(\alpha_{1},\alpha_{2}\right)$ for each order of kinematic expansion. For this purpose, the corresponding vector can be conveniently introduced

(35) $$S^{\left(\tau \right)}={\left[\begin{array}{ccccccccc} N_{1}^{\left(\tau \right)} & N_{2}^{\left(\tau \right)} & N_{12}^{\left(\tau \right)} & N_{21}^{\left(\tau \right)} & T_{1}^{\left(\tau \right)} & T_{2}^{\left(\tau \right)} & P_{1}^{\left(\tau \right)} & P_{2}^{\left(\tau \right)} & S_{3}^{\left(\tau \right)} \end{array}\right]}^{T}$$

for τ=0,1,2,…,N,N+1. The stress resultant in hand is defined by the following matrix notation

(36) $$S^{\left(\tau \right)}=\sum_{\eta =0}^{N+1}A^{\left(\tau \eta \right)}\varepsilon^{\left(\eta \right)}$$

where the stiffness matrix of the laminate $A^{\left(\tau \eta \right)}$ is given by

(37) $$A^{\left(\tau \eta \right)}=\sum_{k=1}^{l}\int_{\zeta_{k}}^{\zeta_{k+1}}{\left(Z^{\left(\tau \right)}\right)}^{T}{\bar{C}}^{\left(k\right)}Z^{\left(\eta \right)}H_{1}H_{2}d\zeta$$

for τ,η=0,1,2,…,N,N+1. As mentioned above, the definitions and the meaning of higher-order stress resultants are illustrated in the book by Tornabene et al. [1].

The constitutive operator $A^{\left(\tau \eta \right)}$ is a 9×9 matrix that assumes the following aspect

(38) $$A^{\left(\tau \eta \right)}=\left[\begin{array}{ccccccccc} A_{11\left(20\right)}^{\left(\tau \eta \right)} & A_{12\left(11\right)}^{\left(\tau \eta \right)} & A_{16\left(20\right)}^{\left(\tau \eta \right)} & A_{16\left(11\right)}^{\left(\tau \eta \right)} & 0 & 0 & 0 & 0 & A_{13\left(10\right)}^{\left(\tau \tilde{\eta }\right)}\\ A_{12\left(11\right)}^{\left(\tau \eta \right)} & A_{22\left(02\right)}^{\left(\tau \eta \right)} & A_{26\left(11\right)}^{\left(\tau \eta \right)} & A_{26\left(02\right)}^{\left(\tau \eta \right)} & 0 & 0 & 0 & 0 & A_{23\left(01\right)}^{\left(\tau \tilde{\eta }\right)}\\ A_{16\left(20\right)}^{\left(\tau \eta \right)} & A_{26\left(11\right)}^{\left(\tau \eta \right)} & A_{66\left(20\right)}^{\left(\tau \eta \right)} & A_{66\left(11\right)}^{\left(\tau \eta \right)} & 0 & 0 & 0 & 0 & A_{36\left(10\right)}^{\left(\tau \tilde{\eta }\right)}\\ A_{16\left(11\right)}^{\left(\tau \eta \right)} & A_{26\left(02\right)}^{\left(\tau \eta \right)} & A_{66\left(11\right)}^{\left(\tau \eta \right)} & A_{66\left(02\right)}^{\left(\tau \eta \right)} & 0 & 0 & 0 & 0 & A_{36\left(01\right)}^{\left(\tau \tilde{\eta }\right)}\\ 0 & 0 & 0 & 0 & A_{44\left(20\right)}^{\left(\tau \eta \right)} & A_{45\left(11\right)}^{\left(\tau \eta \right)} & A_{44\left(10\right)}^{\left(\tau \tilde{\eta }\right)} & A_{45\left(10\right)}^{\left(\tau \tilde{\eta }\right)} & 0\\ 0 & 0 & 0 & 0 & A_{45\left(11\right)}^{\left(\tau \eta \right)} & A_{55\left(02\right)}^{\left(\tau \eta \right)} & A_{45\left(01\right)}^{\left(\tau \tilde{\eta }\right)} & A_{55\left(01\right)}^{\left(\tau \tilde{\eta }\right)} & 0\\ 0 & 0 & 0 & 0 & A_{44\left(10\right)}^{\left(\tilde{\tau }\eta \right)} & A_{45\left(01\right)}^{\left(\tilde{\tau }\eta \right)} & A_{44\left(00\right)}^{\left(\tilde{\tau }\tilde{\eta }\right)} & A_{45\left(00\right)}^{\left(\tilde{\tau }\tilde{\eta }\right)} & 0\\ 0 & 0 & 0 & 0 & A_{45\left(10\right)}^{\left(\tilde{\tau }\eta \right)} & A_{55\left(01\right)}^{\left(\tilde{\tau }\eta \right)} & A_{45\left(00\right)}^{\left(\tilde{\tau }\tilde{\eta }\right)} & A_{55\left(00\right)}^{\left(\tilde{\tau }\tilde{\eta }\right)} & 0\\ A_{13\left(10\right)}^{\left(\tilde{\tau }\eta \right)} & A_{23\left(01\right)}^{\left(\tilde{\tau }\eta \right)} & A_{36\left(10\right)}^{\left(\tilde{\tau }\eta \right)} & A_{36\left(01\right)}^{\left(\tilde{\tau }\eta \right)} & 0 & 0 & 0 & 0 & A_{33\left(00\right)}^{\left(\tilde{\tau }\tilde{\eta }\right)} \end{array}\right]$$

In extended notation, relation (36) can be written as follows for a generic HSDT whose maximum order of expansion is given by N (embedded with the Murakami’s function)

(39) $$\left[\begin{array}{c} S^{\left(0\right)}\\ S^{\left(1\right)}\\ \vdots \\ \vdots \\ S^{\left(N\right)}\\ S^{\left(N+1\right)} \end{array}\right]=\left[\begin{array}{cccccc} A^{\left(00\right)} & A^{\left(01\right)} & \cdots & \cdots & A^{\left(0\left(N\right)\right)} & A^{\left(0\left(N+1\right)\right)}\\ A^{\left(10\right)} & A^{\left(11\right)} & \cdots & \cdots & A^{\left(1\left(N\right)\right)} & A^{\left(1\left(N+1\right)\right)}\\ \vdots & \vdots & \ddots & & \vdots & \vdots \\ \vdots & \vdots & & \ddots & \vdots & \vdots \\ A^{\left(\left(N\right)0\right)} & A^{\left(\left(N\right)1\right)} & \cdots & \cdots & A^{\left(\left(N\right)\left(N\right)\right)} & A^{\left(\left(N\right)\left(N+1\right)\right)}\\ A^{\left(\left(N+1\right)0\right)} & A^{\left(\left(N+1\right)1\right)} & \cdots & \cdots & A^{\left(\left(N+1\right)\left(N\right)\right)} & A^{\left(\left(N+1\right)\left(N+1\right)\right)} \end{array}\right]\left[\begin{array}{c} \varepsilon^{\left(0\right)}\\ \varepsilon^{\left(1\right)}\\ \vdots \\ \vdots \\ \varepsilon^{\left(N\right)}\\ \varepsilon^{\left(N+1\right)} \end{array}\right]$$

With reference to the well-known FSDT, the following correspondences can be deduced

(40) $$A^{\left(00\right)}=A,\;A^{\left(01\right)}=B,\;A^{\left(10\right)}=B,\;A^{\left(11\right)}=D$$

in which A,D,B are classically known as membrane stiffness matrix, bending stiffness matrix, and coupling stiffness matrix [1]. In fact, τ=0 and τ=1 are related respectively to the membrane stresses and bending moments (analogously for the strains), denoted by $S^{\left(0\right)}$ and $S^{\left(1\right)}$ ($\varepsilon^{\left(0\right)}$ and $\varepsilon^{\left(1\right)}$ as far as the generalized strains are concerned). On the other hand, for τ≥2, relations among higher-order stresses and strains exist. Thus, higher-order terms could be coupled or uncoupled according to the mechanical properties of the structure (lamination scheme), following the same principles of the FSDT. For instance, it is well-known that the elements in B assume zero values for symmetric laminates. The same feature is repeated also for higher-order coupling terms. Each element inside the matrix (38) can be evaluated as follows

(41) $$\begin{array}{l} A_{nm\;\left(pq\right)}^{\left(\tau \eta \right)}=\sum_{k=1}^{l}\int_{\zeta_{k}}^{\zeta_{k+1}}{\bar{B}}_{nm}^{\left(k\right)}F_{\eta }^{}F_{\tau }^{}\frac{H_{1}H_{2}}{H_{1}^{p}H_{2}^{q}}d\zeta \\ A_{nm\;\left(pq\right)}^{\left(\tilde{\tau }\eta \right)}=\sum_{k=1}^{l}\int_{\zeta_{k}}^{\zeta_{k+1}}{\bar{B}}_{nm}^{\left(k\right)}F_{\eta }^{}\frac{\partial F_{\tau }^{}}{\partial \zeta }\frac{H_{1}H_{2}}{H_{1}^{p}H_{2}^{q}}d\zeta \\ A_{nm\;\left(pq\right)}^{\left(\tau \tilde{\eta }\right)}=\sum_{k=1}^{l}\int_{\zeta_{k}}^{\zeta_{k+1}}{\bar{B}}_{nm}^{\left(k\right)}\frac{\partial F_{\eta }^{}}{\partial \zeta }F_{\tau }^{}\frac{H_{1}H_{2}}{H_{1}^{p}H_{2}^{q}}d\zeta \\ A_{nm\;\left(pq\right)}^{\left(\tilde{\tau }\tilde{\eta }\right)}=\sum_{k=1}^{l}\int_{\zeta_{k}}^{\zeta_{k+1}}{\bar{B}}_{nm}^{\left(k\right)}\frac{\partial F_{\eta }^{}}{\partial \zeta }\frac{\partial F_{\tau }^{}}{\partial \zeta }\frac{H_{1}H_{2}}{H_{1}^{p}H_{2}^{q}}d\zeta \end{array}$$

for τ,η=0,1,2,…,N,N+1, n,m=1,2,3,4,5,6, and p,q=0,1,2. It should be highlighted that the considerations just illustrated, in particular the correspondences in (40), are valid if the power-law function $\zeta^{\;\tau }$ is taken as thickness functions. For a different choice of thickness functions, in fact, definitions (41) provide stiffness matrices that are different from the classic ones (A,D,B) related to first-order terms. The symbols ${\bar{B}}_{nm}^{\left(k\right)}\left(\alpha_{1},\alpha_{2}\right)$ in (41) stand for the elastic coefficients of the k-th layer. They can be computed as shown below

(42) $$\begin{array}{l} {\bar{B}}_{nm}^{\left(k\right)}={\bar{E}}_{nm}^{\left(k\right)}\;\mathrm{for}\;n,m=1,2,3,6\\ {\bar{B}}_{nm}^{\left(k\right)}=\frac{{\bar{E}}_{nm}^{\left(k\right)}}{\chi }\;\mathrm{for}\;n,m=4,5 \end{array}$$

in which the shear correction factor is identified by χ. Such distinction is essential to introduce the possibility to correct the distributions of the shear stresses. In fact, HSDTs in general do not require this coefficient. Nevertheless, in the present paper this correction is applied to the structural models up to the second order of kinematic expansion. Further details concerning the use of the shear correction factor in higher-order theories can be found in the works [93,94,95]. Some comments related to this correction are presented also in the next sections. Finally, it should be specified that integrals in (41) must be solved numerically. As illustrated in the previous paper by the authors [95], the Generalized Integral Quadrature (GIQ) method represents an accurate numerical tool to deal with this kind of issue. Classic integration schemes could be used as well [2].

By means of Hamilton’s variational principle [1,4], a set of three equilibrium equations are obtained for each order of kinematic expansion τ. The structures are loaded only on their external surfaces by normal or shear pressures. These applied forces per unit surface are denoted by $q_{1}^{\left(+\right)},\;q_{2}^{\left(+\right)},\;q_{3}^{\left(+\right)}$ and $q_{1}^{\left(-\right)},\;q_{2}^{\left(-\right)},\;q_{3}^{\left(-\right)}$. It should be noted that the superscript ^(+)^ specifies that the top surface is loaded, whereas the superscript ^(−)^ is used to identify a load applied on the other external surface. On the other hand, the subscript represents the coordinate along which the force is applied. The static equivalence principle is employed to evaluate the generalized load components acting on the shell middle surface, given by $q_{1}^{\left(\tau \right)},\;q_{2}^{\left(\tau \right)},\;q_{3}^{\left(\tau \right)}$, which can be collected in the corresponding vector

(43) $$q^{\left(\tau \right)}={\left[\begin{array}{ccc} q_{1}^{\left(\tau \right)} & q_{2}^{\left(\tau \right)} & q_{3}^{\left(\tau \right)} \end{array}\right]}^{T}$$

The following expressions can be used to define each quantity in (43) for each order of kinematic expansion

(44) $$\begin{array}{l} q_{1}^{\left(\tau \right)}=q_{1}^{\left(-\right)}F_{\tau }^{\left(-\right)}H_{1}^{\left(-\right)}H_{2}^{\left(-\right)}+q_{1}^{\left(+\right)}F_{\tau }^{\left(+\right)}H_{1}^{\left(+\right)}H_{2}^{\left(+\right)}\\ q_{2}^{\left(\tau \right)}=q_{2}^{\left(-\right)}F_{\tau }^{\left(-\right)}H_{1}^{\left(-\right)}H_{2}^{\left(-\right)}+q_{2}^{\left(+\right)}F_{\tau }^{\left(+\right)}H_{1}^{\left(+\right)}H_{2}^{\left(+\right)}\\ q_{3}^{\left(\tau \right)}=q_{3}^{\left(-\right)}F_{\tau }^{\left(-\right)}H_{1}^{\left(-\right)}H_{2}^{\left(-\right)}+q_{3}^{\left(+\right)}F_{\tau }^{\left(+\right)}H_{1}^{\left(+\right)}H_{2}^{\left(+\right)} \end{array}$$

where $H_{1}^{\left(\pm \right)},\;H_{2}^{\left(\pm \right)}$, and $F_{\tau }^{\left(\pm \right)}$ represent the values that both the geometric quantities in (6) and the thickness functions assumes on the shell external surfaces (for ζ=±h/2). At this point, the equilibrium equations are carried out for τ=0,1,2,…,N,N+1

(45) $$D_{\Omega }^{*}S^{\left(\tau \right)}+q^{\left(\tau \right)}=0$$

where the operator $D_{\Omega }^{*}$ is given by

(46) $$D_{\Omega }^{*}={\left[\begin{array}{ccc} \frac{1}{A_{1}}\frac{\partial }{\partial \alpha_{1}}+\frac{1}{A_{1}A_{2}}\frac{\partial A_{2}}{\partial \alpha_{1}} & -\frac{1}{A_{1}A_{2}}\frac{\partial A_{\;1}}{\partial \alpha_{2}} & -\frac{1}{R_{\;1}}\\ -\frac{1}{A_{1}A_{2}}\frac{\partial A_{\;2}}{\partial \alpha_{1}} & \frac{1}{A_{2}}\frac{\partial }{\partial \alpha_{2}}+\frac{1}{A_{1}A_{2}}\frac{\partial A_{1}}{\partial \alpha_{2}} & -\frac{1}{R_{\;2}}\\ \frac{1}{A_{1}A_{2}}\frac{\partial A_{1}}{\partial \alpha_{2}} & \frac{1}{A_{1}}\frac{\partial }{\partial \alpha_{1}}+\frac{1}{A_{1}A_{2}}\frac{\partial A_{2}}{\partial \alpha_{1}} & 0\\ \frac{1}{A_{2}}\frac{\partial }{\partial \alpha_{2}}+\frac{1}{A_{1}A_{2}}\frac{\partial A_{1}}{\partial \alpha_{2}} & \frac{1}{A_{1}A_{2}}\frac{\partial A_{2}}{\partial \alpha_{1}} & 0\\ \frac{1}{R_{\;1}} & 0 & \frac{1}{A_{1}}\frac{\partial }{\partial \alpha_{1}}+\frac{1}{A_{1}A_{2}}\frac{\partial A_{2}}{\partial \alpha_{1}}\\ 0 & \frac{1}{R_{\;2}} & \frac{1}{A_{2}}\frac{\partial }{\partial \alpha_{2}}+\frac{1}{A_{1}A_{2}}\frac{\partial A_{1}}{\partial \alpha_{2}}\\ -1 & 0 & 0\\ 0 & -1 & 0\\ 0 & 0 & -1 \end{array}\right]}^{T}$$

By means of relations (36) and (15), the equilibrium equations can be written as a function of the generalized displacements of the middle surface

(47) $$\sum_{\eta =0}^{N+1}L^{\left(\tau \eta \right)}u^{\left(\eta \right)}+q^{\left(\tau \right)}=0$$

where the fundamental operator $L^{\left(\tau \eta \right)}$ is given by the following 3×3 matrix

(48) $$L^{\left(\tau \eta \right)}=D_{\Omega }^{*}A^{\left(\tau \eta \right)}D_{\Omega }^{}$$

for τ,η=0,1,2,…,N,N+1. A proper set of boundary conditions must be introduced to solve this system of partial differential equations. For the sake of conciseness, the boundary conditions are summarized in Table 1 for the external edges of the shell element depicted in Figure 1. In the following sections, the boundary conditions are specified by following the order “WSEN”, where the four letters stand for the lateral edges of the shell (Figure 1). For instance, “SSSS” and “CCCC” mean that all the four edges are simply-supported and clamped, respectively. On the other hand, “FCFC” is used to define a structure with the western and eastern edges free, whereas the others are clamped. A shell with only the northern edge free is specified as “CCCF”. Finally, due to the considerable number of variables and definitions, a nomenclature section is included in Appendix A for the sake of clarity.

## 4. Numerical Solution

**Figure 3:**
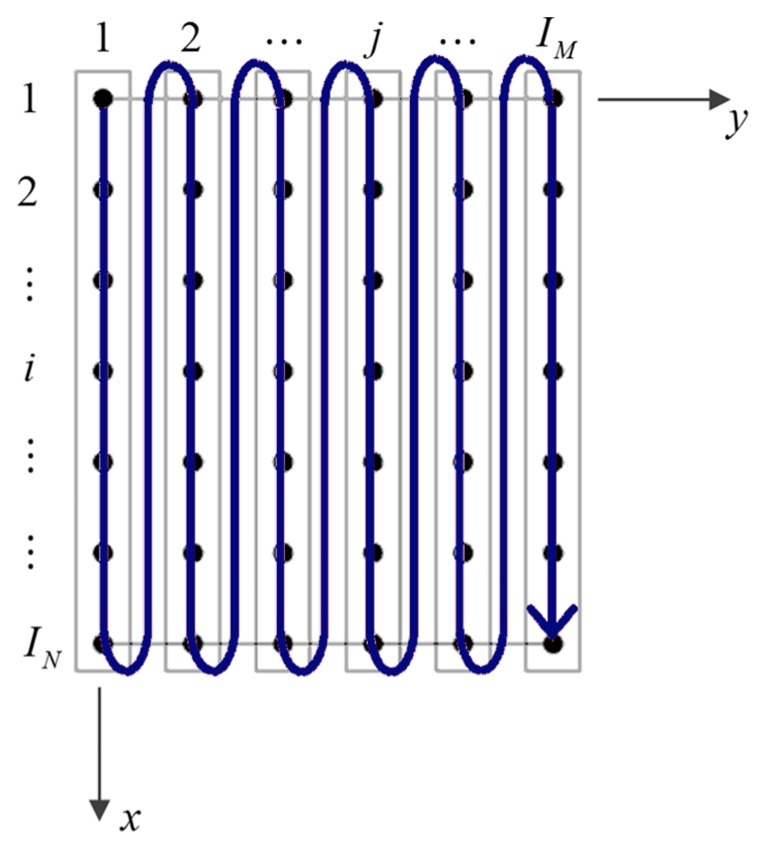
Grid point order for the implementation of the Generalized Differential Quadrature (GDQ) technique within a two-dimensional domain [2].

The GDQ technique is used to obtain and solve the discrete form of the governing equations. Indeed, the present numerical method provides the solution of the strong formulation of the fundamental Equation (47) by approximating the partial derivatives. For completeness purposes, the main aspects of this technique are presented briefly [131]. Let us consider a two-dimensional domain in which $I_{N},\;I_{M}$ denote respectively the total number of discrete points along x and y, where x,y represents the principal coordinates of the domain itself. The n-th order derivative with respect to x, the m-th order derivative with respect to y, and the n+m order mixed derivative of a generic function f(x,y) evaluated at the discrete point $\left(x_{i},y_{j}\right)$ are given by

(49) $${f_{x}^{\left(n\right)}\left(x_{i},y_{j}\right)=\frac{\partial^{\left(n\right)}f\left(x,y\right)}{\partial x^{\left(n\right)}}|}_{x=x_{i},y=y_{j}}=\sum_{k=1}^{I_{N}}\varsigma_{x\left(ik\right)}^{\left(n\right)}f_{\left(kj\right)}$$

(50) $$f_{y}^{\left(m\right)}\left(x_{i},y_{j}\right)={\frac{\partial^{\left(m\right)}f\left(x,y\right)}{\partial y^{\left(m\right)}}|}_{x=x_{i},y=y_{j}}=\sum_{l=1}^{I_{M}}\varsigma_{y\left(jl\right)}^{\left(m\right)}f_{\left(il\right)}$$

(51) $${f_{xy}^{\left(n+m\right)}\left(x_{i},y_{j}\right)=\frac{\partial^{\left(n+m\right)}f\left(x,y\right)}{\partial x^{\left(n\right)}\partial y^{\left(m\right)}}|}_{x=x_{i},y=y_{j}}=\sum_{k=1}^{I_{N}}\varsigma_{x\left(ik\right)}^{\left(n\right)}\left(\sum_{l=1}^{I_{M}}\varsigma_{y\left(jl\right)}^{\left(m\right)}f_{\left(kl\right)}\right)$$

where the notation $f\left(x_{i},y_{j}\right)=f_{\left(ij\right)}$ is employed. The weighting coefficients are specified by $\varsigma_{x\left(ik\right)}^{\left(n\right)}$ and $\varsigma_{y\left(jl\right)}^{\left(m\right)}$. They can be evaluated by using the Lagrange polynomials. For conciseness purpose, only the definition of the coefficients related to the first coordinate x is given here. For the first-order derivatives, one gets

(52) $$\varsigma_{x\left(ik\right)}^{\left(1\right)}=\frac{L^{\;\left(1\right)}\left(x_{i}\right)}{\left(x_{i}-x_{k}\right)L^{\;\left(1\right)}\left(x_{k}\right)}$$

whereas the weighting coefficients for higher-order derivatives can be computed recursively as

(53) $$\varsigma_{x\left(ik\right)}^{\left(n\right)}=n\left(\varsigma_{x\left(ii\right)}^{\left(n-1\right)}\varsigma_{x\left(ik\right)}^{\left(1\right)}-\frac{\varsigma_{x\left(ik\right)}^{\left(n-1\right)}}{x_{i}-x_{k}}\right)\;\mathrm{for}\;i\neq k$$

(54) $$\varsigma_{x\left(ii\right)}^{\left(n\right)}=-\sum_{k=1,k\neq i}^{I_{N}}\varsigma_{x\left(ik\right)}^{\left(n\right)}\;\mathrm{for}\;i=k$$

assuming $i,k=1,2,\ldots ,I_{N}$. The Lagrange polynomials are denoted by L(x) and they are defined as follows

(55) $$L\left(x\right)=\prod_{j=1}^{I_{N}}\left(x-x_{j}\right)$$

from which the corresponding first-order derivatives $L^{\;\left(1\right)}\left(x_{i}\right)$ can be deducted and evaluated at the point $x_{i}$. The weighting coefficients $\varsigma_{y\left(jl\right)}^{\left(m\right)}$ can be computed following the same procedure. The weighting coefficients $\varsigma_{x\left(ik\right)}^{\left(n\right)}$ and $\varsigma_{y\left(jl\right)}^{\left(m\right)}$ can be conveniently collected in the corresponding matrices $\varsigma_{x}^{\left(n\right)}$ and $\varsigma_{y}^{\left(n\right)}$, whose size is given by $I_{N}\times I_{N}$ and $I_{M}\times I_{M}$, respectively. A simplified approach for the implementation of this technique can be used by collecting the grid points of the domain as specified by the blue arrow in Figure 3.

Thus, the order of the point is given by $\left(x_{1},y_{1}\right)$, $\left(x_{2},y_{1}\right)$, …, $\left(x_{I_{N}},y_{1}\right)$, $\left(x_{1},y_{2}\right)$, …, $\left(x_{I_{N}},y_{2}\right)$, $\left(x_{1},y_{I_{M}}\right)$, …, $\left(x_{I_{N}},y_{I_{M}}\right)$. The values that the generic function f(x,y) assume in each point are also collected by following the same order, so that the following algebraic vector f is obtained

(56) $$\begin{array}{l} f=[\underset{}{}\underset{\mathrm{first}\;\mathrm{column}}{\underset{︸}{\begin{array}{cccc} f{\left(x_{1},y_{1}\right)}_{1} & f{\left(x_{2},y_{1}\right)}_{2} & \ldots & f{\left(x_{I_{N}},y_{1}\right)}_{I_{N}} \end{array}}}\ldots \ldots \\ \;\ldots \ldots \underset{\mathrm{second}\;\mathrm{column}}{\underset{︸}{\begin{array}{ccc} f{\left(x_{1},y_{2}\right)}_{I_{N}+1} & \ldots & f{\left(x_{I_{N}},y_{2}\right)}_{2I_{N}} \end{array}}}\ldots \ldots \\ \;\ldots \ldots \underset{\mathrm{last}\;\mathrm{column}}{\underset{︸}{\begin{array}{ccc} f{\left(x_{1},y_{I_{M}}\right)}_{I_{N}\cdot I_{M}-I_{N}+1} & \ldots & f{\left(x_{I_{N}},y_{I_{M}}\right)}_{I_{N}\cdot I_{M}} \end{array}}}{\underset{}{}]}^{T} \end{array}$$

where $f_{k}=f{\left(x_{i},y_{j}\right)}_{k}$, for $i=1,2,\ldots ,I_{N}$, $j=1,2,\ldots ,I_{M}$ and $k=i+\left(j-1\right)I_{N}$. The size of f is given by $\left(I_{N}\cdot I_{M}\right)\times 1$. The weighting coefficients can be computed as

(57) $$\underset{\left(I_{N}\cdot I_{M}\right)\times \left(I_{N}\cdot I_{M}\right)}{C_{x}^{\left(n\right)}}=\underset{I_{M}\times I_{M}}{\;I_{}}⊗\underset{I_{N}\times I_{N}}{\varsigma_{x}^{\left(n\right)}}$$

(58) $$\underset{\left(I_{N}\cdot I_{M}\right)\times \left(I_{N}\cdot I_{M}\right)}{C_{y}^{\left(m\right)}}=\underset{I_{M}\times I_{M}}{\varsigma_{y}^{\left(m\right)}}⊗\underset{I_{N}\times I_{N}}{\;I_{}}$$

(59) $$\underset{\left(I_{N}\cdot I_{M}\right)\times \left(I_{N}\cdot I_{M}\right)}{C_{xy}^{\left(n+m\right)}}=\underset{I_{M}\times I_{M}}{\varsigma_{y}^{\left(m\right)}}⊗\underset{I_{N}\cdot I_{N}}{\varsigma_{x}^{\left(n\right)}}$$

where I is the identity matrix, whereas the symbol ⊗ stands for the Kronecker product. The size of each operator in (57)–(59) is specified under the various matrices. $C_{x}^{\left(n\right)}$, $C_{y}^{\left(m\right)}$, and $C_{xy}^{\left(n+m\right)}$ are the weighting coefficients matrices that allow the corresponding derivatives in each point of the domain to be computed as simple matrix products

(60) $$f_{x}^{\left(n\right)}=C_{x}^{\left(n\right)}f$$

(61) $$f_{y}^{\left(m\right)}=C_{y}^{\left(m\right)}f$$

(62) $$f_{xy}^{\left(n+m\right)}=C_{xy}^{\left(n+m\right)}f$$

where $f_{x}^{\left(n\right)}$, $f_{y}^{\left(m\right)}$, and $f_{xy}^{\left(n+m\right)}$ represent the vectors of size $\left(I_{N}\cdot I_{M}\right)\times 1$ which collect the derivatives of the function f(x,y) within the whole domain, following the same order employed in (56). It should be highlighted that this approach does not set any restriction on the choice of the grid distribution. The points can be placed within the domain following different schemes. In the present paper, the discrete points of the shell reference surface are given by

(63) $$\alpha_{1i}=\frac{\alpha_{1}^{1}-\alpha_{1}^{0}}{r_{I_{N}}-r_{1}}\left(r_{i}-r_{1}\right)+\alpha_{1}^{0}$$

(64) $$\alpha_{2j}=\frac{\alpha_{2}^{1}-\alpha_{2}^{0}}{r_{I_{M}}-r_{1}}\left(r_{j}-r_{1}\right)+\alpha_{2}^{0}$$

where the meaning of $r_{i}$ and $r_{j}$, for $i=1,2,\ldots ,I_{N}$ and $j=1,2,\ldots ,I_{M}$, is specified in Table 2 for various distributions.

## 5. Solution of the Static Problem

The static solution of the problem is obtained applying the GDQ technique, which allows the following discrete form of the governing equations to be written

(65) Kδ=f

where K is the stiffness matrix of size $\left(3\times \left(N+2\right)\times \left(I_{N}\times I_{M}\right)\right)\times \left(3\times \left(N+2\right)\times \left(I_{N}\times I_{M}\right)\right)$, δ the displacement vector, and f the external load vector. Both δ and f are given by a vector characterized by the same size $\left(3\times \left(N+2\right)\times \left(I_{N}\times I_{M}\right)\right)\times 1$. It should be noted that the degrees of freedom of the problem are ordered following the scheme shown in Figure 3 for each order of kinematic expansion. By means of the so-called static condensation procedure, the size of the problem can be reduced by separating the degrees of freedom linked to the inner points of the domain (*d*) from the ones related to the boundary nodes (*b*). As a consequence, all the quantities in (65) are evaluated to consider this classification as follows

(66) $$\left[\begin{array}{cc} K_{bb} & K_{bd}\\ K_{db} & K_{dd} \end{array}\right]\left[\begin{array}{c} \delta_{b}\\ \delta_{d} \end{array}\right]=\left[\begin{array}{c} f_{b}\\ f_{d} \end{array}\right]$$

The generalized displacements of the boundary points $\delta_{b}$ are easily obtained as

(67) $$\delta_{b}=K_{bb}^{-1}\left(f_{b}-K_{bd}\delta_{d}\right)$$

whereas the generalized displacements collected in $\delta_{d}$ can be deduced from the following relation

(68) $$\left(K_{dd}-K_{db}K_{bb}^{-1}K_{bd}\right)\delta_{d}=f_{d}-K_{db}K_{bb}^{-1}\;f_{b}$$

It can be observed that the size of the problem turns out to be $3\times \left(N+2\right)\times \left(\left(I_{N}-2\right)\times \left(I_{M}-2\right)\right)$, which represents a reduced value if compared to the original size of the problem shown in (65). Finally, it should be specified that the derivatives needed to compute the stress resultants (36) involved in the boundary conditions for free or simply-supported edges (Table 1) are computed through the GDQ method.

## 6. Strain and Stress Recovery Procedure

Once the static solution is obtained, the three-dimensional elasticity equations in terms of stresses can be used to evaluate the through-the-thickness variation of all these quantities. In fact, the three indefinite equilibrium equations of elasticity written in an orthogonal curvilinear coordinate system allows the three-dimensional profiles of stresses and strains to be obtained, consequently, even if the higher-order structural model illustrated above is two-dimensional. The following treatise presents the main aspects of the recovery procedure at issue. Once the generalized displacements of the middle surface are computed, the kinematic model (10) is employed to obtain the three-displacements of the solid medium in each point $\alpha_{1}{}_{i},\alpha_{2}{}_{j}$ of the reference surface

(69) $$\begin{array}{l} U_{1\left(ijm\right)}\left(\alpha_{1i},\alpha_{2j},\zeta_{m}\right)=\sum_{\tau =0}^{N+1}F_{\tau \left(m\right)}^{}\left(\zeta_{m}\right)u_{1\left(ij\right)}^{\left(\tau \right)}\left(\alpha_{1i},\alpha_{2j}\right)\\ U_{2\left(ijm\right)}\left(\alpha_{1i},\alpha_{2j},\zeta_{m}\right)=\sum_{\tau =0}^{N+1}F_{\tau \left(m\right)}^{}\left(\zeta_{m}\right)u_{2\left(ij\right)}^{\left(\tau \right)}\left(\alpha_{1i},\alpha_{2j}\right)\\ U_{3\left(ijm\right)}\left(\alpha_{1i},\alpha_{2j},\zeta_{m}\right)=\sum_{\tau =0}^{N+1}F_{\tau \left(m\right)}^{{}_{}}\left(\zeta_{m}\right)u_{3\left(ij\right)}^{\left(\tau \right)}\left(\alpha_{1i},\alpha_{2j}\right) \end{array}$$

for $i\;=\;1,\;2,\ldots ,\;I_{N}$, $j\;=\;1,\;2,\ldots ,\;I_{M}$, and $m=1,2,\ldots ,I_{T}$, where $I_{T}$ denotes the number of discrete points along the thickness for each ply of the laminate. The Cheb-Gau-Lob distribution with $I_{T}=25$ is used to obtain a discrete distribution of points $\zeta_{m}$ along the thickness of each layer

(70) $$\zeta_{m}=\frac{\zeta_{k+1}-\zeta_{k}}{2}\left(1+\cos\left(\frac{I_{T}-m}{I_{T}-1}\pi \right)\right)+\zeta_{k}$$

for $m=1,2,\ldots ,I_{T}$. It should be specified that $F_{\tau \left(m\right)}^{}$ represents the value that the corresponding thickness function assumes at the point $\zeta_{m}$.

By means of the GDQ technique, the generalized strains (15) can be computed in each grid point. As a consequence, the three-dimensional strain components related to each discrete point of the middle surface can be evaluated by using expression (18). The strains at issue assume the following aspect

(71) $$\varepsilon_{\left(ijm\right)}={\left[\begin{array}{cccccc} \varepsilon_{1\left(ijm\right)} & \varepsilon_{2\left(ijm\right)} & \gamma_{12\left(ijm\right)} & \gamma_{13\left(ijm\right)} & \gamma_{23\left(ijm\right)} & \varepsilon_{n\left(ijm\right)} \end{array}\right]}^{T}$$

In particular, quantities $\varepsilon_{1\left(ijm\right)},\;\varepsilon_{2\left(ijm\right)},\;\gamma_{12\left(ijm\right)}$ collected in (71) are required to obtain the membrane stresses $\sigma_{1\left(ijm\right)}$, $\sigma_{2\left(ijm\right)}$, and $\tau_{12\left(ijm\right)}$ in each point of the domain, by means of the constitutive relations (21). The three-dimensional equilibrium equations along the principal curvilinear coordinates are presented below

(72) $$\begin{array}{l} \frac{\partial \tau_{1n}}{\partial \zeta }+\tau_{1n}\left(\frac{2}{R_{\;1}+\zeta }+\frac{1}{R_{\;2}+\zeta }\right)=-\frac{1}{A_{\;1}\left(1+\zeta /R_{\;1}\right)}\frac{\partial \sigma_{1}}{\partial \alpha_{1}}+\frac{\sigma_{2}-\sigma_{1}}{A_{\;1}A_{\;2}\left(1+\zeta /R_{\;2}\right)}\frac{\partial A_{\;2}}{\partial \alpha_{1}}+\\ \;-\frac{1}{A_{\;2}\left(1+\zeta /R_{\;2}\right)}\frac{\partial \tau_{12}}{\partial \alpha_{2}}-\frac{2\tau_{12}}{A_{\;1}A_{\;2}\left(1+\zeta /R_{\;1}\right)}\frac{\partial A_{\;1}}{\partial \alpha_{2}} \end{array}$$

(73) $$\begin{array}{l} \frac{\partial \tau_{2n}}{\partial \zeta }+\tau_{2n}\left(\frac{1}{R_{\;1}+\zeta }+\frac{2}{R_{\;2}+\zeta }\right)=-\frac{1}{A_{\;2}\left(1+\zeta /R_{\;2}\right)}\frac{\partial \sigma_{2}}{\partial \alpha_{2}}+\frac{\sigma_{1}-\sigma_{2}}{A_{\;1}A_{\;2}\left(1+\zeta /R_{\;1}\right)}\frac{\partial A_{\;1}}{\partial \alpha_{2}}+\\ \;-\frac{1}{A_{\;1}\left(1+\zeta /R_{\;1}\right)}\frac{\partial \tau_{12}}{\partial \alpha_{1}}-\frac{2\tau_{12}}{A_{\;1}A_{\;2}\left(1+\zeta /R_{\;2}\right)}\frac{\partial A_{\;2}}{\partial \alpha_{1}} \end{array}$$

(74) $$\begin{array}{l} \frac{\partial \sigma_{n}}{\partial \zeta }+\sigma_{n}\left(\frac{1}{R_{\;1}+\zeta }+\frac{1}{R_{\;2}+\zeta }\right)=-\frac{1}{A_{\;1}\left(1+\zeta /R_{\;1}\right)}\frac{\partial \tau_{1n}}{\partial \alpha_{1}}-\frac{\tau_{1n}}{A_{\;1}A_{\;2}\left(1+\zeta /R_{\;2}\right)}\frac{\partial A_{\;2}}{\partial \alpha_{1}}+\\ \;-\frac{1}{A_{\;2}\left(1+\zeta /R_{\;2}\right)}\frac{\partial \tau_{2n}}{\partial \alpha_{2}}-\frac{\tau_{2n}}{A_{\;1}A_{\;2}\left(1+\zeta /R_{\;1}\right)}\frac{\partial A_{\;1}}{\partial \alpha_{2}}\;+\frac{\sigma_{1}}{R_{\;1}+\zeta }+\frac{\sigma_{2}}{R_{\;2}+\zeta } \end{array}$$

The GDQ method is employed to approximate the partial derivatives of the membrane stresses as follows

(75) $$\begin{array}{l} {\frac{\partial \sigma_{1}^{}}{\partial \alpha_{1}}|}_{\left(ijm\right)}≅\sum_{k=1}^{I_{\text{​}N}}\varsigma_{ik}^{\alpha_{1}\left(1\right)}\sigma_{1\left(kjm\right)},\;{\frac{\partial \sigma_{2}^{}}{\partial \alpha_{2}}|}_{\left(ijm\right)}≅\sum_{k=1}^{I_{\text{​}M}}\varsigma_{jk}^{\alpha_{2}\left(1\right)}\sigma_{2\left(ikm\right)}\\ {\frac{\partial \tau_{12}^{}}{\partial \alpha_{1}}|}_{\left(ijm\right)}≅\sum_{k=1}^{I_{\text{​}N}}\varsigma_{ik}^{\alpha_{1}\left(1\right)}\tau_{12\left(kjm\right)},\;{\frac{\partial \tau_{12}^{}}{\partial \alpha_{2}}|}_{\left(ijm\right)}≅\sum_{k=1}^{I_{\text{​}M}}\varsigma_{jk}^{\alpha_{2}\left(1\right)}\tau_{12\left(ikm\right)} \end{array}$$

where $\varsigma_{ik}^{\alpha_{1}\left(1\right)},\varsigma_{jk}^{\alpha_{2}\left(1\right)}$ represents the weighting coefficients for the derivatives within the shell middle surface. The discrete form of the equilibrium Equations (72)–(74) is obtained numerically through the GDQ technique as well

(76) $$\begin{array}{l} \sum_{k=1}^{I_{T}}\varsigma_{mk}^{\zeta \left(1\right)}\tau_{1n\left(ijk\right)}+\tau_{1n\left(ijm\right)}\left(\frac{2}{R_{\;1\left(ij\right)}+\zeta_{m}}+\frac{1}{R_{\;2\left(ij\right)}+\zeta_{m}}\right)=\\ \;=-\frac{1}{A_{\;1\left(ij\right)}\left(1+\zeta_{m}/R_{\;1\left(ij\right)}\right)}{\frac{\partial \sigma_{1}}{\partial \alpha_{1}}|}_{\left(ijm\right)}+\frac{\sigma_{2\left(ijm\right)}-\sigma_{1\left(ijm\right)}}{A_{\;1\left(ij\right)}A_{\;2\left(ij\right)}\left(1+\zeta_{m}/R_{\;2\left(ij\right)}\right)}{\frac{\partial A_{\;2}}{\partial \alpha_{1}}|}_{\left(ij\right)}+\\ \;-\frac{1}{A_{\;2\left(ij\right)}\left(1+\zeta_{m}/R_{\;2\left(ij\right)}\right)}{\frac{\partial \tau_{12}}{\partial \alpha_{2}}|}_{\left(ijm\right)}-\frac{2\tau_{12\left(ijm\right)}}{A_{\;1\left(ij\right)}A_{\;2\left(ij\right)}\left(1+\zeta_{m}/R_{\;1\left(ij\right)}\right)}{\frac{\partial A_{\;1}}{\partial \alpha_{2}}|}_{\left(ij\right)} \end{array}$$

(77) $$\begin{array}{l} \sum_{k=1}^{I_{T}}\varsigma_{mk}^{\zeta \left(1\right)}\tau_{2n\left(ijk\right)}+\tau_{2n\left(ijm\right)}\left(\frac{1}{R_{\;1\left(ij\right)}+\zeta_{m}}+\frac{2}{R_{\;2\left(ij\right)}+\zeta_{m}}\right)=\\ \;=-\frac{1}{A_{\;2\left(ij\right)}\left(1+\zeta_{m}/R_{\;2\left(ij\right)}\right)}{\frac{\partial \sigma_{2}}{\partial \alpha_{2}}|}_{\left(ijm\right)}+\frac{\sigma_{1\left(ijm\right)}-\sigma_{2\left(ijm\right)}}{A_{\;1\left(ij\right)}A_{\;2\left(ij\right)}\left(1+\zeta_{m}/R_{\;1\left(ij\right)}\right)}{\frac{\partial A_{\;1}}{\partial \alpha_{2}}|}_{\left(ij\right)}+\\ \;-\frac{1}{A_{\;1\left(ij\right)}\left(1+\zeta_{m}/R_{\;1\left(ij\right)}\right)}{\frac{\partial \tau_{12}}{\partial \alpha_{1}}|}_{\left(ijm\right)}-\frac{2\tau_{12\left(ijm\right)}}{A_{\;1\left(ij\right)}A_{\;2\left(ij\right)}\left(1+\zeta_{m}/R_{\;2\left(ij\right)}\right)}{\frac{\partial A_{\;2}}{\partial \alpha_{1}}|}_{\left(ij\right)} \end{array}$$

(78) $$\begin{array}{l} \sum_{k=1}^{I_{\text{​}T}}\varsigma_{mk}^{\zeta \left(1\right)}\sigma_{n\left(ijk\right)}+\sigma_{n\left(ijm\right)}\left(\frac{1}{R_{\;1\left(ij\right)}+\zeta_{m}}+\frac{1}{R_{\;2\left(ij\right)}+\zeta_{m}}\right)=\\ \;=\frac{\sigma_{1\left(ijm\right)}}{R_{\;1\left(ij\right)}+\zeta_{m}}+\frac{\sigma_{2\left(ijm\right)}}{R_{\;2\left(ij\right)}+\zeta_{m}}-\frac{1}{A_{\;1\left(ij\right)}\left(1+\zeta_{m}/R_{\;1\left(ij\right)}\right)}{\frac{\partial \tau_{1n}}{\partial \alpha_{1}}|}_{\left(ijm\right)}-\frac{\tau_{1n\left(ijm\right)}}{A_{\;1\left(ij\right)}A_{\;2\left(ij\right)}\left(1+\zeta_{m}/R_{\;2\left(ij\right)}\right)}{\frac{\partial A_{\;2}}{\partial \alpha_{1}}|}_{\left(ij\right)}+\\ \;-\frac{1}{A_{\;2\left(ij\right)}\left(1+\zeta_{m}/R_{\;2\left(ij\right)}\right)}{\frac{\partial \tau_{2n}}{\partial \alpha_{2}}|}_{\left(ijm\right)}-\frac{\tau_{2n\left(ijm\right)}}{A_{\;1\left(ij\right)}A_{\;2\left(ij\right)}\left(1+\zeta_{m}/R_{\;1\left(ij\right)}\right)}{\frac{\partial A_{\;1}}{\partial \alpha_{2}}|}_{\left(ij\right)} \end{array}$$

in which $\varsigma_{mk}^{\zeta \left(1\right)}$ are the weighting coefficients for the derivatives along ζ. The Lamè parameters $A_{\;1\left(ij\right)},A_{\;2\left(ij\right)}$ and the radii of curvature $R_{\;1\left(ij\right)},R_{\;2\left(ij\right)}$ are also computed in each discrete point of the reference surface. Relations (76) and (77), which must be written for each layer of the laminate, allow the shear stresses $\tau_{1n},\tau_{2n}$ to be obtained by imposing the boundary conditions on the bottom surface of the structure

(79) $$\begin{array}{l} {\bar{\tau }}_{1n\left(ij1\right)}={\bar{q}}_{1\left(ij\right)}^{\left(-\right)}\\ {\bar{\tau }}_{2n\left(ij1\right)}={\bar{q}}_{2\left(ij\right)}^{\left(-\right)} \end{array}$$

where ${\bar{q}}_{1\left(ij\right)}^{\left(-\right)},{\bar{q}}_{2\left(ij\right)}^{\left(-\right)}$ denote the shear forces applied at the bottom surface. The actual through-the-thickness profiles of the shear stresses in hand can be computed by enforcing a second couple of boundary condition at the top surface of the upper layer of the laminated shell

(80) $$\begin{array}{l} {\bar{\tau }}_{1n\left(ijI_{T}\right)}={\bar{q}}_{1\left(ij\right)}^{\left(+\right)}\\ {\bar{\tau }}_{2n\left(ijI_{T}\right)}={\bar{q}}_{2\left(ij\right)}^{\left(+\right)} \end{array}$$

Conditions (80) are imposed through the following expressions, which represent the effective through-the-thickness variations of the shear stresses in each point of the domain

(81) $$\tau_{1n\left(ijm\right)}={\bar{\tau }}_{1n\left(ijm\right)}+\frac{q_{1\left(ij\right)}^{\left(+\right)}-{\bar{\tau }}_{1n\left(ijI_{T}\right)}}{h}\left(\zeta_{m}+\frac{h}{2}\right)$$

(82) $$\tau_{2n\left(ijm\right)}={\bar{\tau }}_{2n\left(ijm\right)}+\frac{q_{2\left(ij\right)}^{\left(+\right)}-{\bar{\tau }}_{2n\left(ijI_{T}\right)}}{h}\left(\zeta_{m}+\frac{h}{2}\right)$$

for $m=1,2,\ldots ,I_{T}$. It is evident that the symbol $\zeta_{m}$ used in (81) and (82) takes into account the discrete grid distribution applied along each ply, since the correction of the stress components at issue affects the whole laminate thickness. Relations (79) must be seen as interlaminar compatibility conditions when the shear stresses of inner layers are computed. The GDQ method can be applied also to evaluate the derivatives of (81) and (82) with respect to $\alpha_{1},\alpha_{2}$, respectively

(83) $${\frac{\partial \tau_{1n}}{\partial \alpha_{1}}|}_{\left(ijm\right)}≅\sum_{k=1}^{I_{N}}\varsigma_{ik}^{\alpha_{1}\left(1\right)}\tau_{13\left(kjm\right)},\;{\frac{\partial \tau_{2n}}{\partial \alpha_{2}}|}_{\left(ijm\right)}≅\sum_{k=1}^{I_{M}}\varsigma_{jk}^{\alpha_{2}\left(1\right)}\tau_{23\left(ikm\right)}$$

Equation (78) can be now employed to obtain the normal stress by applying the corresponding boundary condition at the bottom surface

(84) $${\bar{\sigma }}_{n\left(ij1\right)}=q_{3\left(ij\right)}^{\left(-\right)}$$

Analogously, another boundary condition must be introduced at the external surface of the top layer

(85) $${\bar{\sigma }}_{n\left(ijI_{T}\right)}=q_{3\left(ij\right)}^{\left(+\right)}$$

where $q_{3\left(ij\right)}^{\left(-\right)},q_{3\left(ij\right)}^{\left(+\right)}$ stand for the applied normal loads on the external surfaces of the shell. Finally, the effective through-the-thickness profile of the normal stress at issue is achieved

(86) $$\sigma_{n\left(ijm\right)}={\bar{\sigma }}_{n\left(ijm\right)}+\frac{q_{n\left(ij\right)}^{\left(+\right)}-{\bar{\sigma }}_{n\left(ijI_{T}\right)}}{h}\left(\zeta_{m}+\frac{h}{2}\right)$$

for $m=1,2,\ldots ,I_{T}$. The same considerations introduced for the previous profiles (81) and (82) are valid also in this circumstance. It should be mentioned that the stresses $\tau_{1n},\tau_{2n},\sigma_{n}$ just computed are continuous at the interfaces among the various layers of the structure. For any point of the three-dimensional solid, the shear strains $\gamma_{1n},\;\gamma_{2n}$ can be evaluated through the constitutive laws (21) as follows

(87) $$\gamma_{1n\;\left(ijm\right)}^{}=\frac{{\bar{C}}_{55}^{\left(m\right)}\tau_{1n\left(ijm\right)}^{}-{\bar{C}}_{45}^{\left(m\right)}\tau_{2n\left(ijm\right)}^{}}{{\bar{C}}_{55}^{\left(m\right)}{\bar{C}}_{44}^{\left(m\right)}-{\left({\bar{C}}_{45}^{\left(m\right)}\right)}^{2}}$$

(88) $$\gamma_{2n\left(ijm\right)}^{}=\frac{{\bar{C}}_{44}^{\left(m\right)}\tau_{2n\left(ijm\right)}^{}-{\bar{C}}_{45}^{\left(m\right)}\tau_{1n\left(ijm\right)}^{}}{{\bar{C}}_{55}^{\left(m\right)}{\bar{C}}_{44}^{\left(m\right)}-{\left({\bar{C}}_{45}^{\left(m\right)}\right)}^{2}}$$

where ${\bar{C}}_{pq}^{\left(m\right)}$ represent the values that the elastic coefficients assume at the m-th discrete point of the layer. The normal strain $\varepsilon_{n}$ is computed by means of the constitutive relations (21) as well

(89) $$\varepsilon_{n\left(ijm\right)}^{}=\frac{\sigma_{n\left(ijm\right)}^{}-{\bar{C}}_{13}^{\left(m\right)}\varepsilon_{1\left(ijm\right)}^{}-{\bar{C}}_{23}^{\left(m\right)}{}_{}\;\varepsilon_{2\left(ijm\right)}^{}-{\bar{C}}_{36}^{\left(m\right)}{}_{}\;\gamma_{12\left(ijm\right)}^{}}{{\bar{C}}_{33}^{\left(m\right)}{}_{}\;}$$

It should be noted that the quantity in (89) is evaluated neglecting the hypothesis of plane stress, thus the non-reduced elastic coefficients are employed. Moreover, the strain components (87)–(89) could be discontinuous at the layer interfaces since no compatibility interlaminar conditions are enforced.

Finally, the actual through-the-thickness variations of the membrane stresses $\sigma_{1},\;\sigma_{2},\;\tau_{12}$ is computed through the constitutive laws (21) by using the effective value of the normal strain $\varepsilon_{n}$. Consequently, the membrane stresses are computed without taking into account the hypothesis of plane stress, even for those structural theories that require that assumption.

## 7. Evaluation of the Elastic Coefficients

As mentioned in the previous sections, the integrals which appear in the definitions of the elastic coefficients $A_{nm\;\left(pq\right)}^{\left(\tau \eta \right)},A_{nm\;\left(pq\right)}^{\left(\tilde{\tau }\eta \right)},A_{nm\;\left(pq\right)}^{\left(\tau \tilde{\eta }\right)},A_{nm\;\left(pq\right)}^{\left(\tilde{\tau }\tilde{\eta }\right)}$ shown in (41) are computed numerically by means of the GIQ method, whose key aspects are summarized here for a one-dimensional domain in which the only variable x is defined in the interval $\left[a_{x},b_{x}\right]$. Let us assume that the reference domain is discretized by $N_{P}$ points. One of the grid distribution of Table 2 could be equally chosen. The integral of a generic function f(x) within the interval $\left[x_{i},x_{j}\right]$, with $x_{i},x_{j}\in \left[a_{x},b_{x}\right]$ is given by

(90) $$\int_{x_{i}}^{x_{j}}f\left(x\right)dx=\sum_{k=1}^{N_{P}}w_{k}^{ij}f\left(x_{k}\right)$$

The procedure to compute the weighting coefficients for the integration $w_{k}^{ij}$ are briefly presented in this paragraph. The following weighting coefficients are required

(91) $$\begin{array}{c} {\bar{\varsigma }}_{x\left(ij\right)}^{\left(1\right)}=\frac{x_{i}-\tilde{c}}{x_{j}-\tilde{c}}\varsigma_{x\left(ij\right)}^{\left(1\right)}\;\mathrm{for}\;i\neq j\\ {\bar{\varsigma }}_{x\left(ij\right)}^{\left(1\right)}=\varsigma_{x\left(ii\right)}^{\left(1\right)}+\frac{1}{x_{i}-\tilde{c}}\;\mathrm{for}\;i=j \end{array}$$

for $i,j=1,2,\ldots ,N_{P}$, where $\varsigma_{x\left(ij\right)}^{\left(1\right)}$ represents the corresponding weighting coefficients for the first-order derivatives that can be easily deducted from Equations (53) and (54). On the other hand, $\tilde{c}$ denotes an arbitrary constant which can be assumed equal to $\tilde{c}=b_{x}+10^{-10}$ for accuracy purposes [131]. The weighting coefficients can be conveniently collected in the corresponding matrix ${\bar{\varsigma }}_{\;x}^{\left(1\right)}$, whose size is given by $N_{P}\times N_{P}$. Its inverse must be computed as

(92) $$W={\left({\bar{\varsigma }}_{\;x}^{\left(1\right)}\right)}^{-1}$$

A generic element of the matrix W is denoted by $w_{ij}^{}$ for $i,j=1,2,\ldots ,N_{P}$. These quantities are used to evaluate the weighting coefficients $w_{k}^{ij}$ for the integral (90) as shown below

(93) $$w_{k}^{ij}=w_{jk}^{}-w_{ik}^{}$$

assuming $k=1,2,\ldots ,N_{P}$, where the indices i,j are related to the boundary points of the interval chosen for the integration. In this paper, the Cheb-Gau-Lob grid distribution is employed in each layer to obtain the quantities in in (41) as presented in (70), with $m=1,2,\ldots ,N_{P}$. The total number of points is set equal to $N_{P}=51$ for each ply.

## 8. Applications

**Figure 4:**
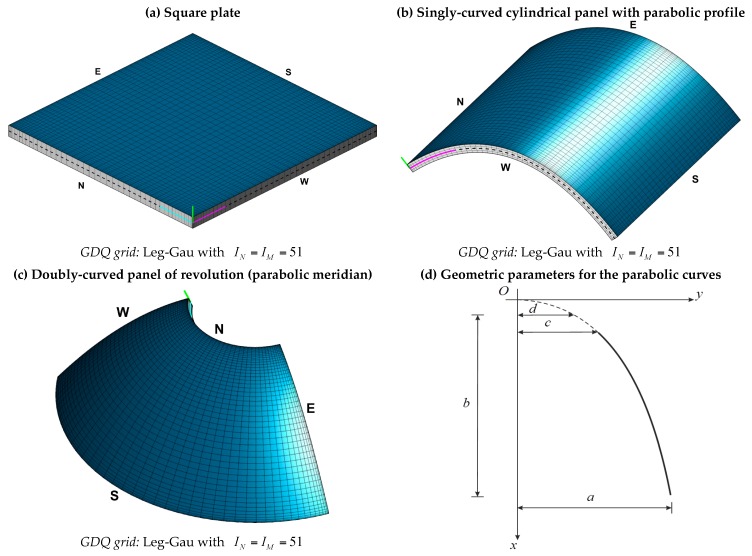
Representation of the geometry of the considered structures: (**a**) Square plate; (**b**) singly-curved cylindrical panel with parabolic profile; (**c**) doubly-curved panel of revolution with parabolic meridian; (**d**) geometric parameters for the parabolic curves. The grid distribution and the number of sampling points required by the GDQ method are also specified.

The present GDQ based approach is implemented in MATLAB code developed by the authors [132], which is employed to achieve the linear static solution of several damaged laminated plates and shells. All the numerical results are shown in this section, which is organized as follows: firstly, a set of convergence analyses in terms of displacements is carried out to prove the stability features of the numerical method for several lamination schemes and HSDTs; then, the recovery procedure is used to obtain the through-the-thickness variations of strain, stress, and displacement components of different structures. For the sake of conciseness, the structural elements are presented in Figure 4, where the employed discrete grid distribution is also specified. In particular, a square plate (Figure 4a), a singly-curved cylindrical panel with parabolic profile (Figure 4b), and a doubly-curved panel of revolution with parabolic meridian (Figure 4c), are considered.

All the mechanical and geometric characteristics of these structures are summarized in Table 3 for brevity purposes. On the other hand, Table 3 provides position vectors, boundary conditions, applied loads, lamination schemes, as well as the points of the domain for the evaluation of the through-the-thickness quantities, for each structural element. On the other hand, the mechanical properties of each constituent are listed in terms of engineering constants in Table 4. Finally, all the functions used for the damage modeling are specified in Table 5, together with all the parameters needed to represent correctly the Gaussian (or elliptic) distributions. Several HSDTs are employed in the following examples. In particular, it should be remarked that the theories up to the second order without the Murakami’s function (${\mathrm{FSDT}}_{RS}^{\chi =1.2}$ and ${\mathrm{ED}2}_{}^{\chi =1.2}$) are always taken with the shear correction factor χ=1.2, whereas the corresponding zig-zag models can be used also without correction for peculiar lamination schemes, as specified in [12,81,93,94,95]. On the other hand, the first-order theories ${\mathrm{FSDT}}_{RS}^{\chi =1.2}$, ${\mathrm{FSDTZ}}_{RS}^{\chi =1.2}$, and ${\mathrm{FSDTZ}}_{RS}^{\chi =1}$, require the hypothesis of plane-stress and the reduced stiffnesses are employed, consequently. In general, the results are presented in terms of displacement variations and through-the-thickness profiles of strains, stresses, and displacements to show the effect of the increase of the damage extent.

### 8.1. Convergence Analyses

**Figure 5:**
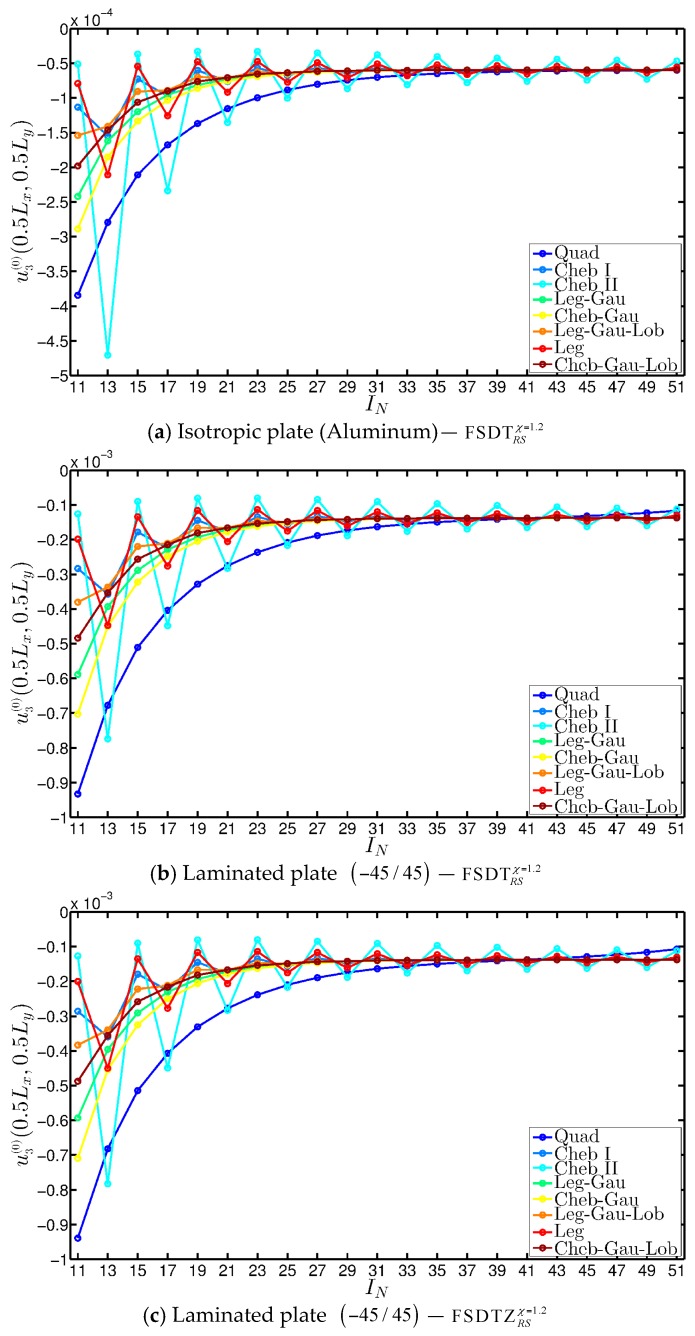
Convergence analysis in terms of central deflection [m] for the SSSS square plate increasing the number of grid points $I_{N}=I_{M}$ for various grid distributions related to the GDQ method. Different mechanical configurations are considered: (**a**) Isotropic (Aluminum); (**b**,**c**) Laminated (Glass-Epoxy) with (−45/45) as stacking sequence. The laminated structure in (b,c) is investigated by means of the ${\mathrm{FSDT}}_{RS}^{\chi =1.2}$ (case b) and the ${\mathrm{FSDTZ}}_{RS}^{\chi =1.2}$ (case c). The same function Gau-1 is applied in each layer to model the damage features.

The convergence analyses are performed for a simply supported (SSSS) square plate (Figure 4a), considering both isotropic and laminated configurations. The ${\mathrm{FSDT}}_{RS}^{\chi =1.2}$ is taken into account for both these two cases, whereas the corresponding theory embedded with the Murakami’s function (${\mathrm{FSDTZ}}_{RS}^{\chi =1.2}$) is also used for the laminated composite, whose stacking sequence is given by (−45/45). The function Gau-1 shown in Table 5 is employed to define the variable mechanical properties of each layer. It should be noted that the convergence analyses are carried out for an extremely concentrated damage characterized by a high deterioration of the mechanical properties. All the grid distributions shown in Table 2 are considered assuming $I_{N}=I_{M}$ and increasing the number of points up to 51. The convergence graphs are presented in Figure 5 in terms of central deflection $u_{3}^{\left(0\right)}\left(0.5L_{x},0.5L_{y}\right)$. Analogously, the numerical values of the same quantity are shown in Table 6 for all the grid distributions.

From the various plots depicted in Figure 5, it can be noted that both the Legendre-Gauss (Leg-Gau) and Chebyshev-Gauss (Cheb-Gau) grid distributions provide a convergent behavior when the static analysis of damaged structures has to be performed. Nevertheless, the Leg-Gau distribution turned out to be the best discrete grid also when the GDQ method is employed to obtain the static response of plates and shells subjected to point or line loads [12]. Since both damages and concentrated forces represent mechanical discontinuities which this approach is able to deal with, the Leg-Gau distribution is used also in this paper.

Finally, it should be highlighted that a good convergence is reached by using many sampling points. Nevertheless, the test at issue is characterized by a noticeable mechanical discontinuity. For this reason, the value $I_{N}=I_{M}=51$ is set in all the following examples independently from the damage features.

### 8.2. Isotropic Square Plate

**Figure 6:**
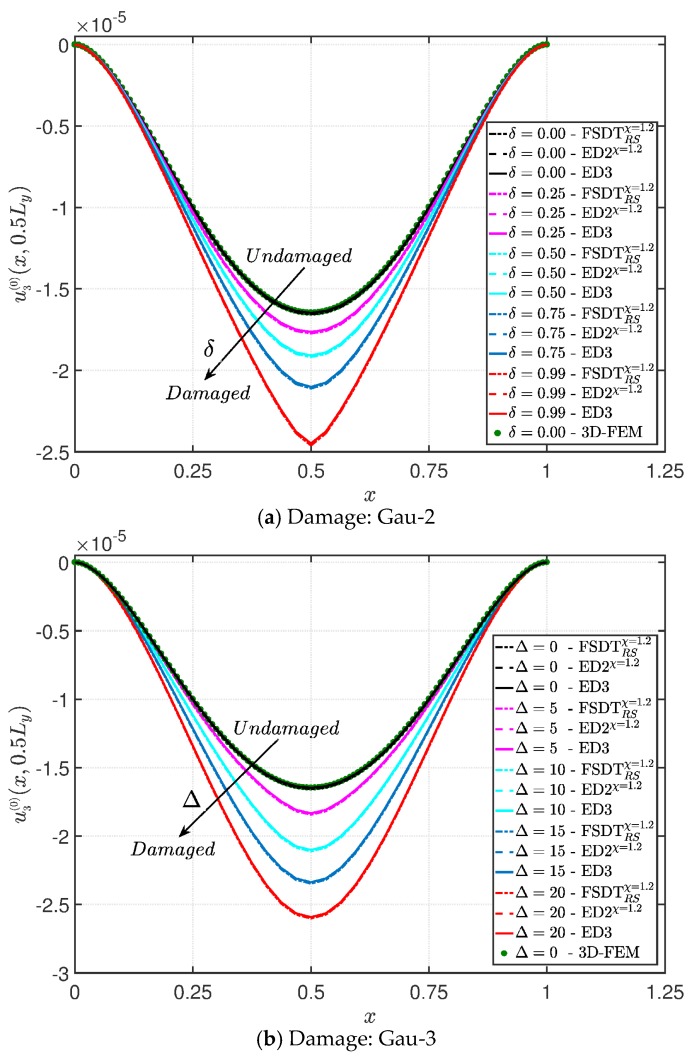
Evaluation of the central vertical displacement profile [m] for the completely clamped boundary conditions (CCCC) isotropic square plate varying the mechanical parameters of the damage: (**a**) Gau-2; (**b**) Gau-3. The results are obtained by means of several Higher-order Shear Deformation Theories (HSDTs).

The same flat structure of the convergence analysis is also used in this application, assuming completely clamped boundary conditions (CCCC). Two damage configurations are considered: Gau-2 and Gau-3. The former provides the increase of damage intensity δ, whereas the latter deals with the extension increase ($\Delta_{1}^{}=\Delta_{2}^{}=\Delta$). The damage is applied in the center of the plate in both these cases ($\alpha_{1m}^{}=\alpha_{2m}^{}=0.5\;m$). The vertical displacement profiles evaluated along the x direction for $y=0.5L_{y}$ are shown in Figure 6a,b, respectively.

**Figure 7:**
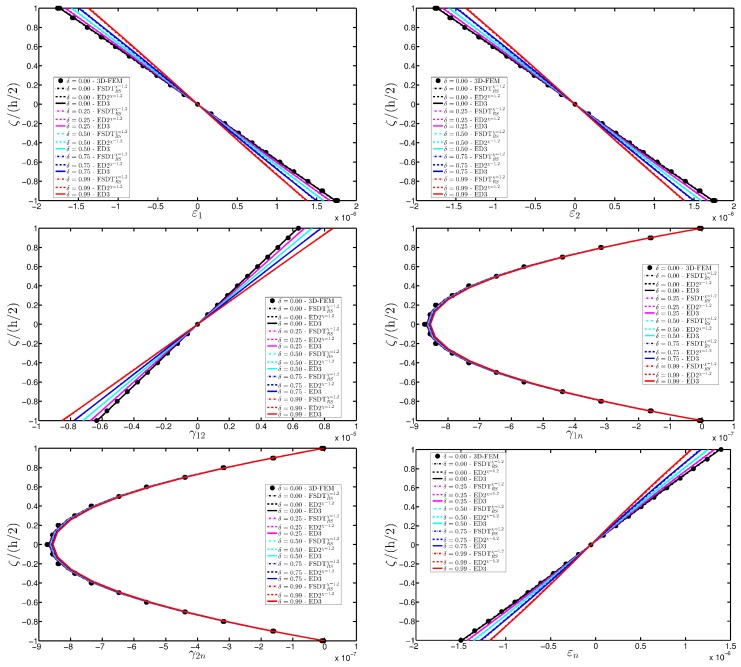
Through-the-thickness profiles of strain components for the CCCC isotropic square plate computed at the point *A* for different HSDTs. The effect of the damage is investigated by varying the parameters of the function Gau-2.

**Figure 8:**
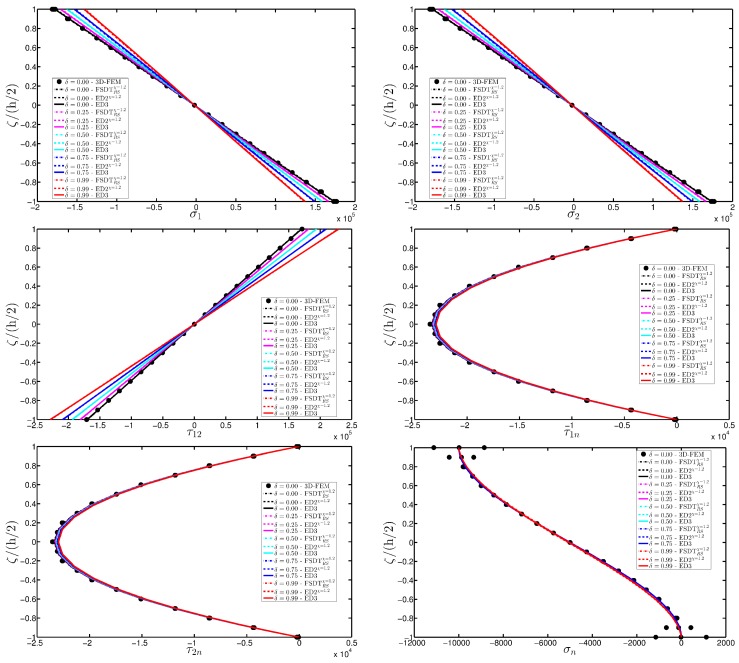
Through-the-thickness profiles of stress components [Pa] for the CCCC isotropic square plate computed at the point *A* for different HSDTs. The effect of the damage is investigated by varying the parameters of the function Gau-2.

**Figure 9:**
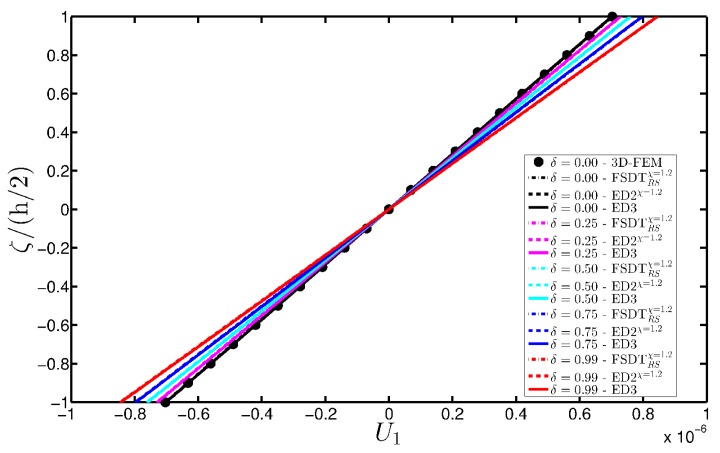

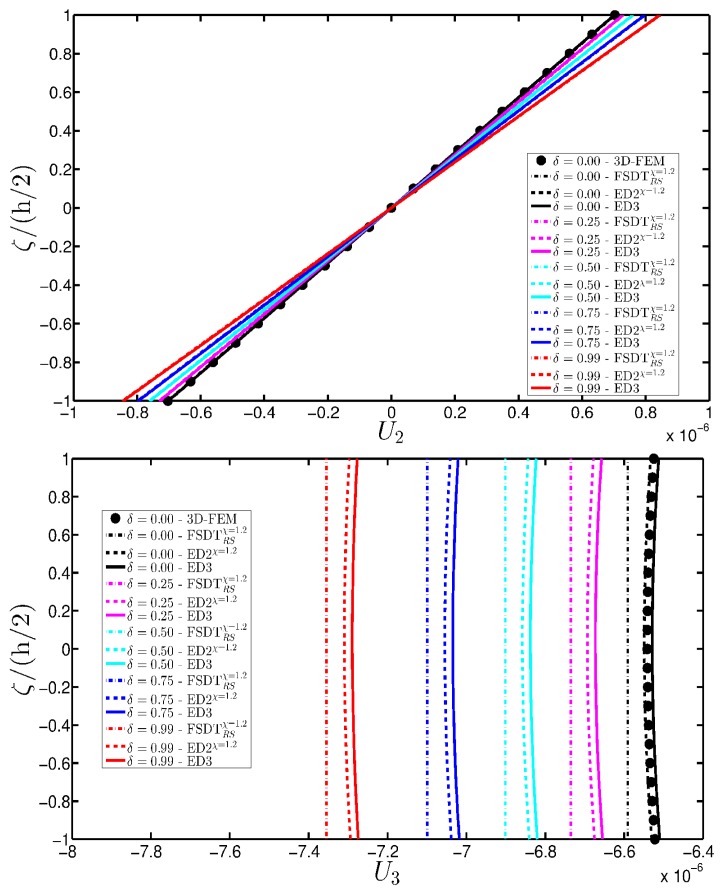
Through-the-thickness profiles of displacement components [m] for the CCCC isotropic square plate computed at the point *A* for different HSDTs. The effect of the damage is investigated by varying the parameters of the function Gau-2.

**Figure 10:**
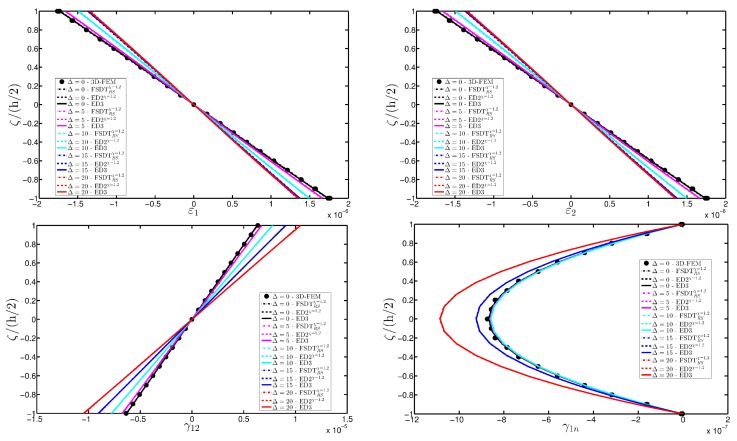

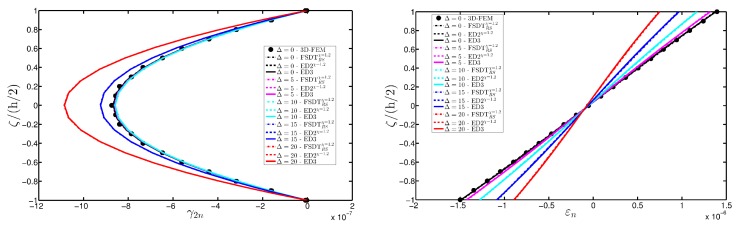
Through-the-thickness profiles of strain components for the CCCC isotropic square plate computed at the point *A* for different HSDTs. The effect of the damage is investigated by varying the parameters of the function Gau-3.

**Figure 11:**
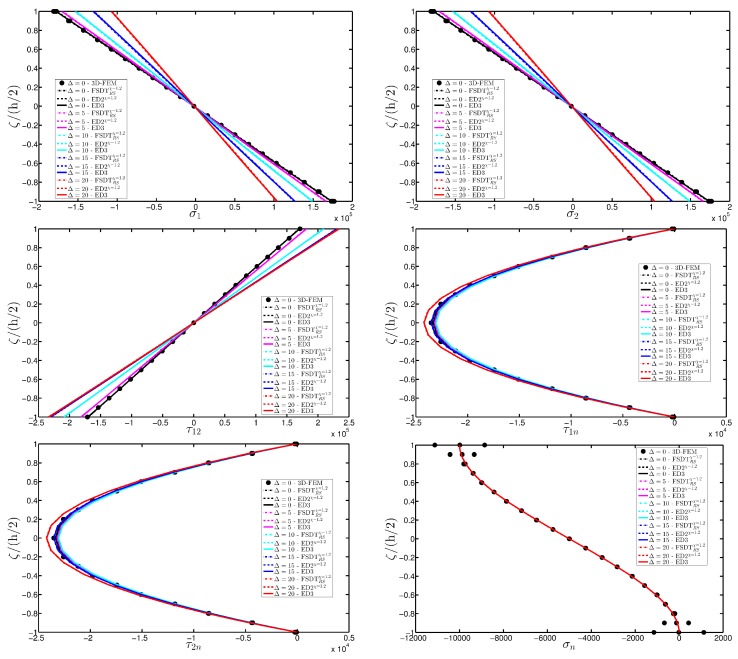
Through-the-thickness profiles of stress components [Pa] for the CCCC isotropic square plate computed at the point A for different HSDTs. The effect of the damage is investigated by varying the parameters of the function Gau-3.

**Figure 12:**
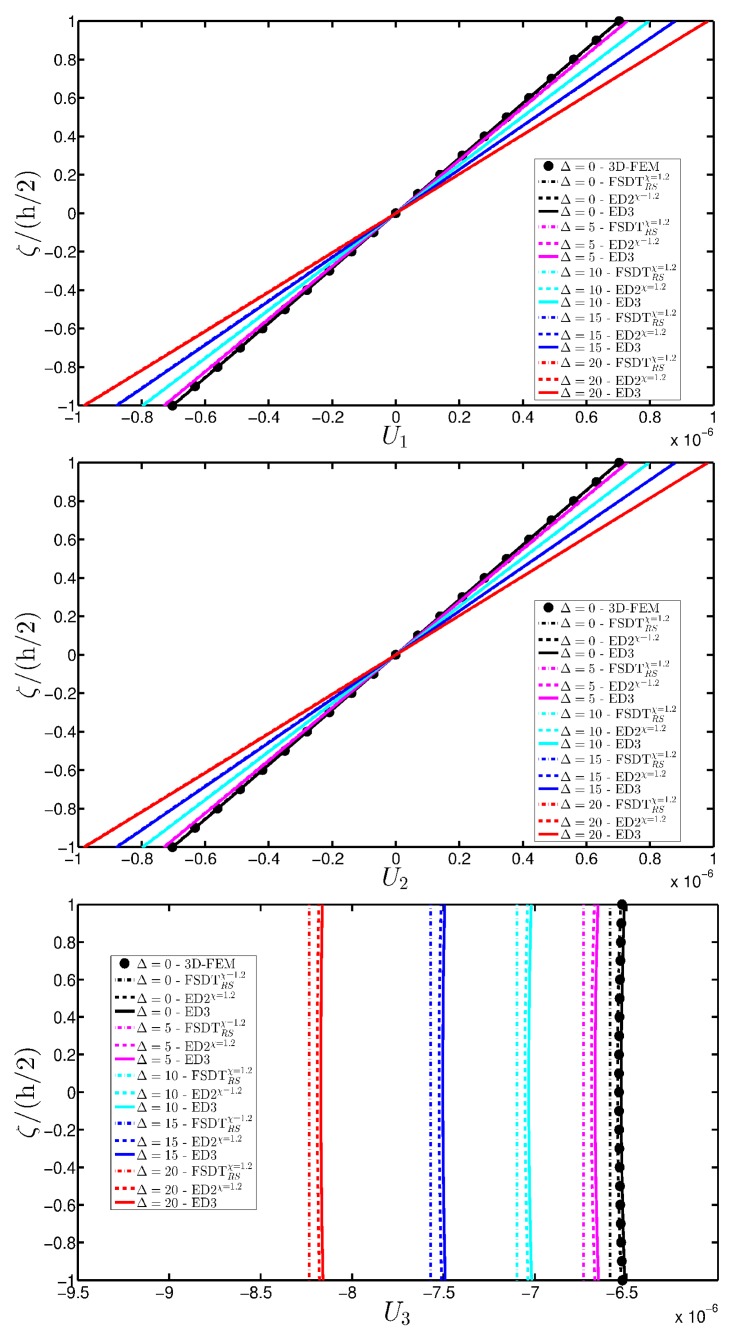
Through-the-thickness profiles of displacement components [m] for the CCCC isotropic square plate computed at the point *A* for different HSDTs. The effect of the damage is investigated by varying the parameters of the function Gau-3.

As expected, the displacements increase when the damage grows or when it involves a bigger area of the domain. The results are compared with the solution obtained with a finite element commercial software (Strand 7) for the undamaged case, by using a three-dimensional model (3D-FEM). The profiles in hand are shown in the graphs through a dotted notation and match perfectly with the corresponding GDQ curves. The through-the-thickness variations of strain, stress, and displacement components are presented in Figure 7, Figure 8 and Figure 9 for the Gau-2 damage model, whereas Figure 10, Figure 11 and Figure 12 deal with the results related to the Gau-3 damage. Even in these circumstances, the 3D-FEM for the undamaged plate is depicted by black dots and it is in good agreement with the present solutions. It can be noted that the damage features can modify the structural response. In particular, displacements are mostly affected by the damage properties. It should be specified that all the HSDTs considered in this application do not require Murakami’s function since the structure is made of a sole isotropic layer. By comparing also the strain profiles related to these cases (shown respectively in Figure 7 and Figure 10), it is possible to observe that a greater extension of damage (keeping the magnitude constant) has more influence on the strain patter than an extremely high decay of mechanical properties although concentrated in a smaller area of the domain. These two circumstances are modeled respectively through Gau-3 and Gau-2 functions.

### 8.3. Laminated Square Plate

**Figure 13:**
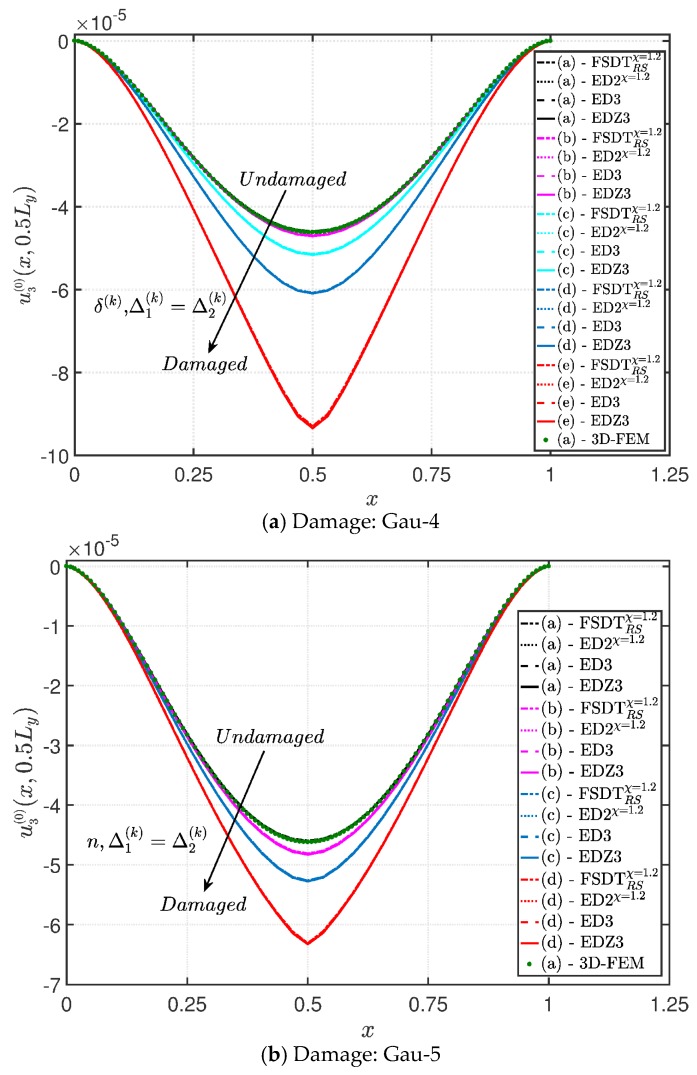
Evaluation of the central vertical displacement profile [m] for the CCCC laminated square plate varying the mechanical parameters of the damage: (**a**) Gau-4; (**b**) Gau-5. The results are obtained by means of several HSDTs.

**Figure 14:**
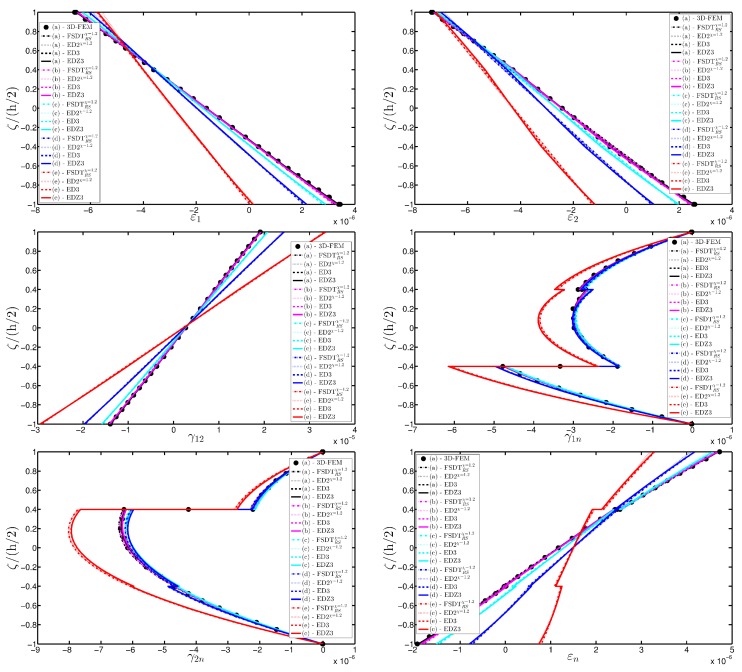
Through-the-thickness profiles of strain components for the CCCC laminated square plate with (−45/0/45) as stacking sequence computed at the point *A* for different HSDTs. The effect of the damage is investigated by varying the parameters of the function Gau-4.

**Figure 15:**
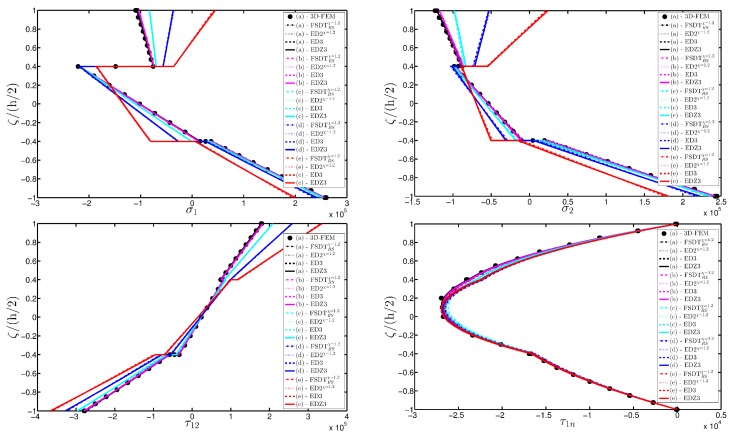

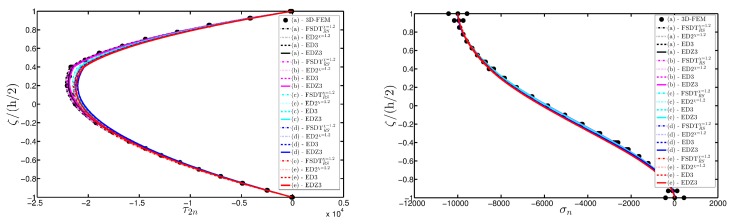
Through-the-thickness profiles of stress components [Pa] for the CCCC laminated square plate with (−45/0/45) as stacking sequence computed at the point *A* for different HSDTs. The effect of the damage is investigated by varying the parameters of the function Gau-4.

**Figure 16:**
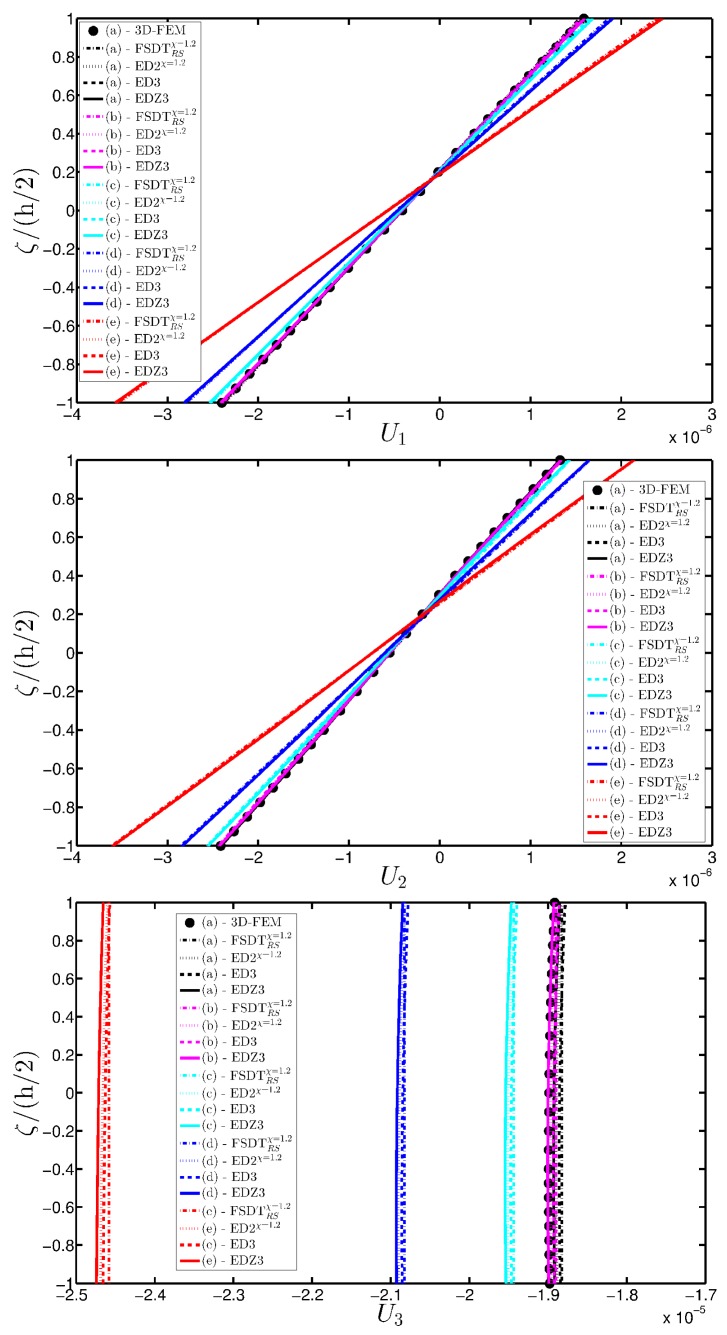
Through-the-thickness profiles of displacement components [m] for the CCCC laminated square plate with (−45/0/45) as stacking sequence computed at the point *A* for different HSDTs. The effect of the damage is investigated by varying the parameters of the function Gau-4.

**Figure 17:**
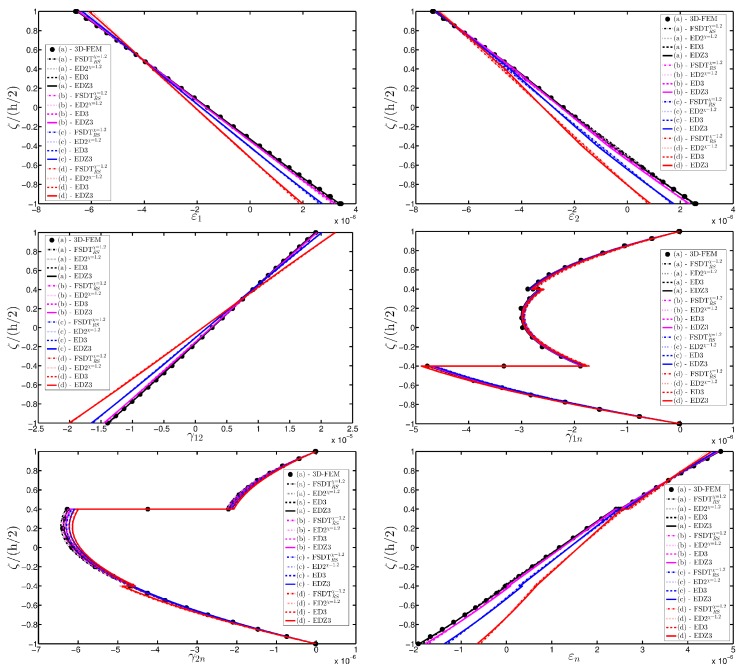
Through-the-thickness profiles of strain components for the CCCC laminated square plate with (−45/0/45) as stacking sequence computed at the point *A* for different HSDTs. The effect of the damage is investigated by varying the parameters of the function Gau-5.

**Figure 18:**
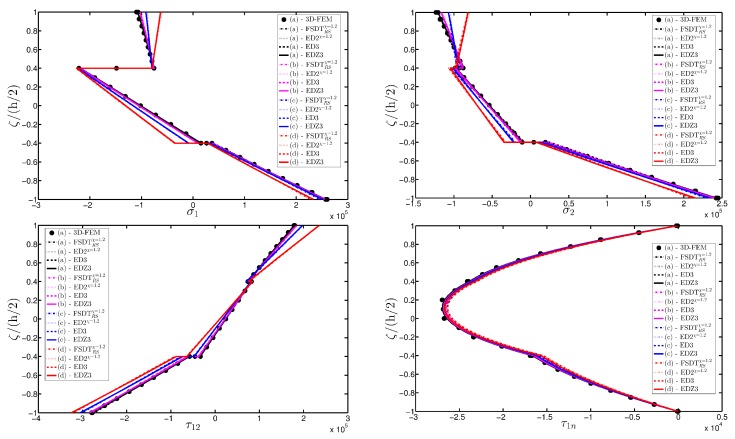

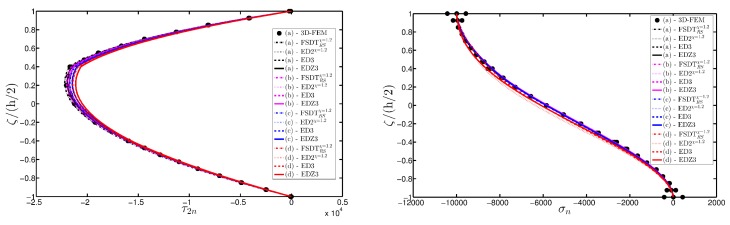
Through-the-thickness profiles of stress components [Pa] for the CCCC laminated square plate with (−45/0/45) as stacking sequence computed at the point *A* for different HSDTs. The effect of the damage is investigated by varying the parameters of the function Gau-5.

**Figure 19:**
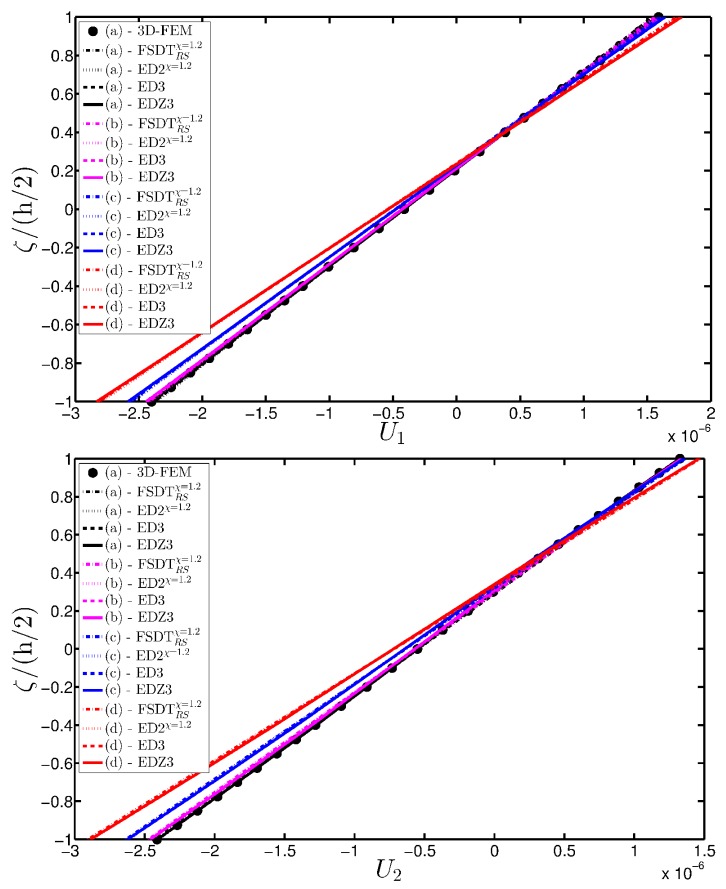

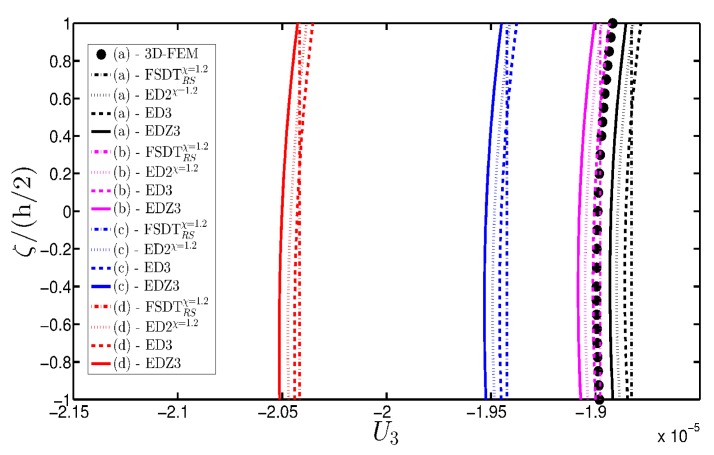
Through-the-thickness profiles of displacement components [m] for the CCCC laminated square plate with (−45/0/45) as stacking sequence computed at the point *A* for different HSDTs. The effect of the damage is investigated by varying the parameters of the function Gau-5.

A completely clamped (CCCC) laminated plate is considered in this paragraph. The geometric features are the same as of the previous test, but the lamination scheme is given by (−45/0/45), in which each layer is made of the same material (see Table 3). Two damage types are considered and they are mathematically described by functions Gau-4 and Gau-5 of Table 4. As in the previous application, the damage is located in the central node of the structure ($\alpha_{1m}^{}=\alpha_{2m}^{}=0.5\;m$). Both the intensity and the size of the deterioration of the mechanical properties are increased in the first case (Gau-4), involving each lamina in the same manner. On the other hand, the damage described by Gau-5 affects the three layers progressively, starting from the bottom ply. In addition, when the damage expands across the upper layers the damaged areas in the lower ones are increased. The graphs of the vertical displacement evaluated along coordinate x with $y=0.5L_{y}$ are depicted in Figure 13a,b for the two damage models, respectively. The 3D-FEM profiles are also shown for comparison purposes. On the other hand, the through-the-thickness variations of strains, stresses, and displacements related to the function Gau-4 are presented in Figure 14, Figure 15 and Figure 16. Figure 17, Figure 18 and Figure 19 show the same quantities for the function Gau-5. The same considerations of the previous example are still valid in this circumstance. It should be noted that similar profiles are obtained by means of the various HSDTs. For completeness purposes, the third-order shear deformation theory is taken with and without the Murakami’s function. No significant differences are observable between the ED3 and the EDZ3.

### 8.4. Cylindrical Panel

A FCFC sandwich structure with a central soft-core is investigated in this paragraph. The shell geometry is given by the singly-curved cylindrical surface with parabolic profile depicted in Figure 4b. The geometric parameters required to represent the surface at issue are listed in Table 3. For the sake of clarity, Figure 4d is added to explain the meaning of the parameters related to the parabolic arch which represents the profile of this cylinder. The same figure can be used as a reference to describe the parabolic meridian of the shell investigated in the following paragraph. A sandwich lamination scheme is considered here. In particular, the mechanical properties of the isotropic soft-core are considerably lower in comparison with the ones of the external orthotropic sheets (Table 3 and Table 4). The damage is modeled through the elliptic function defined as Ell-1 in Table 5.

**Figure 20:**
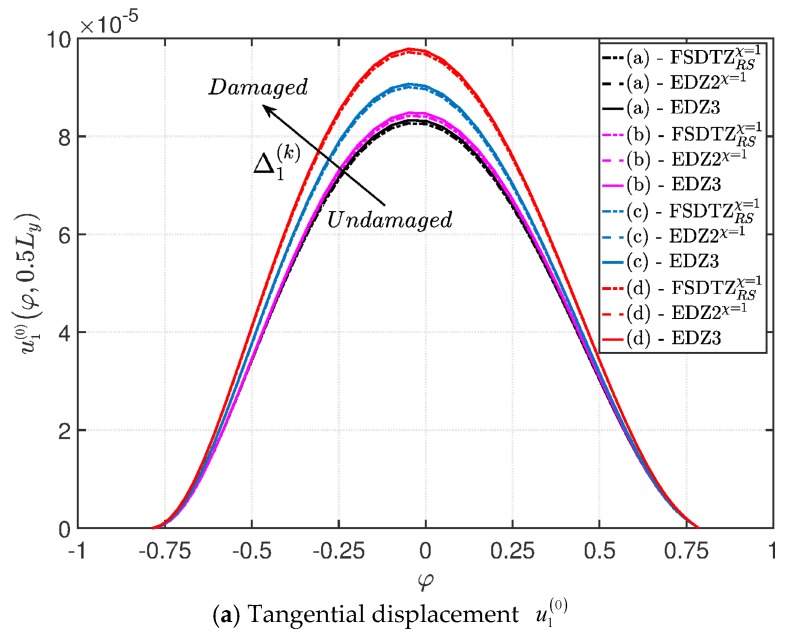

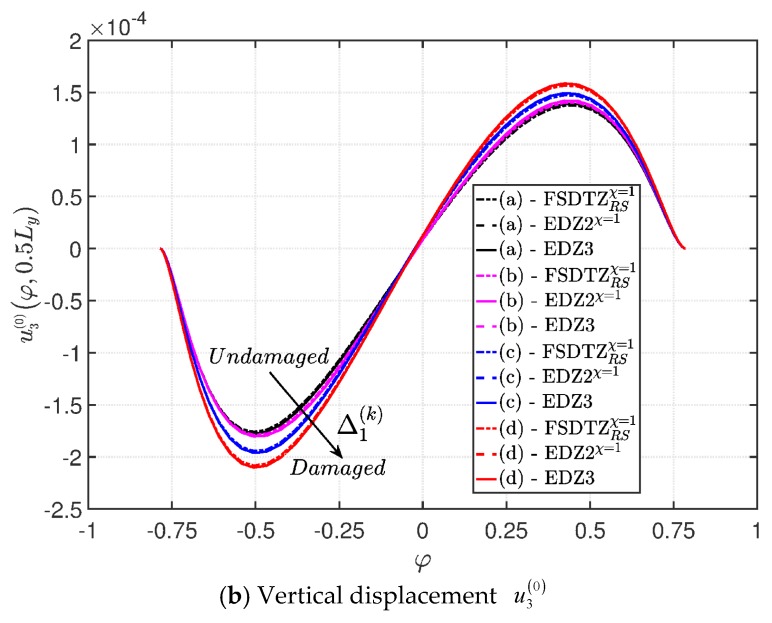
Evaluation of the central displacements profile [m] for the FCFC soft-core cylindrical surface varying the mechanical parameters of the damage (Ell-1): (**a**) Tangential displacement $u_{1}^{\left(0\right)}$; (**b**) Vertical displacement $u_{3}^{\left(0\right)}$. The results are obtained by means of several HSDTs.

**Figure 21:**
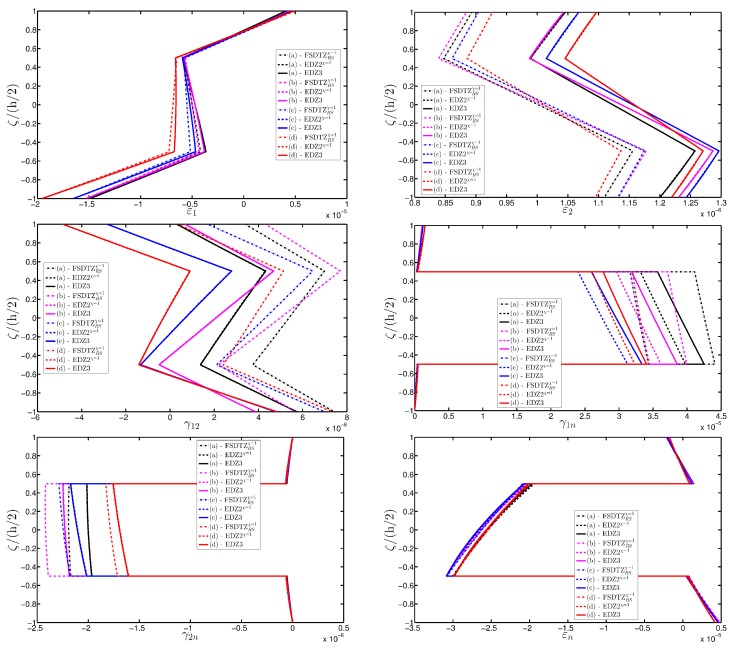
Through-the-thickness profiles of strain components for the FCFC soft-core cylindrical surface computed at the point *B* for different HSDTs. The effect of the damage is investigated by varying the parameters of the function Ell-1.

**Figure 22:**
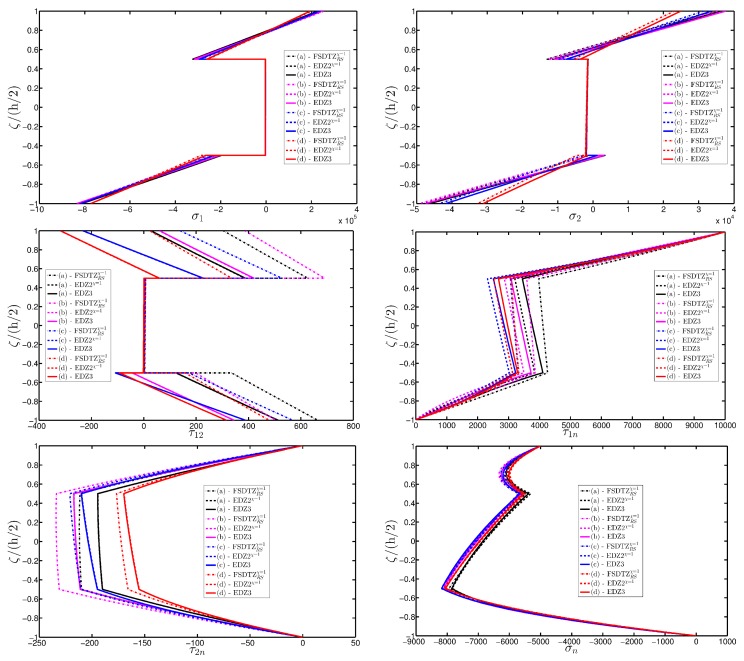
Through-the-thickness profiles of stress components [Pa] for the FCFC soft-core cylindrical surface computed at the point *B* for different HSDTs. The effect of the damage is investigated by varying the parameters of the function Ell-1.

**Figure 23:**
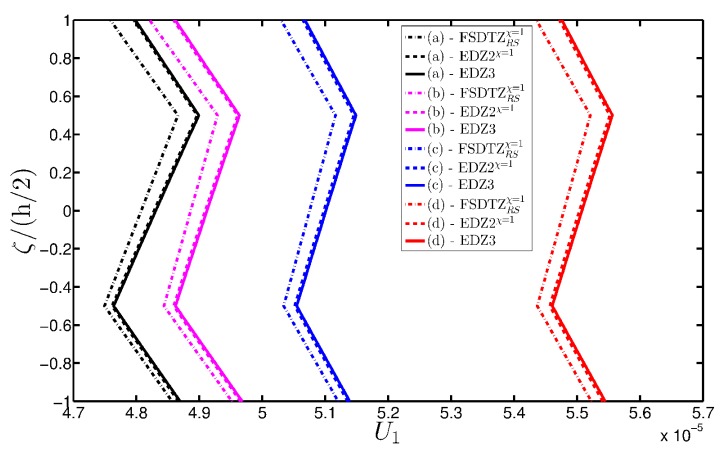

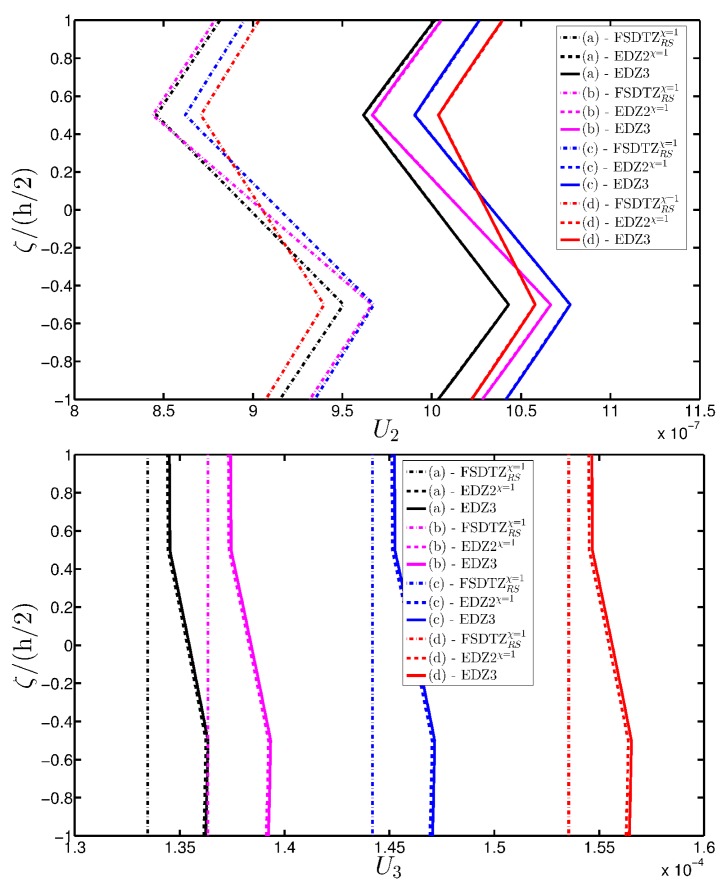
Through-the-thickness profiles of displacement components [m] for the FCFC soft-core cylindrical surface computed at the point *B* for different HSDTs. The effect of the damage is investigated by varying the parameters of the function Ell-1.

The deterioration of the mechanical properties involves only the external layers and the elliptic shape describes a damage that affects the shell along all its length along the coordinate y. The parametric study aims to investigate the effect of a damage that gradually expands itself along the first coordinate φ, as can be noticed from the parameters of the elliptic function Ell-1 in Table 5 ($\Delta_{1}^{\left(k\right)}\ll \Delta_{2}^{\left(k\right)}$, for k=1,3). On the other hand, the intensity of the damage is kept constant. As highlighted in many works [93,94,95], Murakami’s function is needed to deal with this kind of mechanical configuration. Thus, only the corresponding structural theories are considered up to the third-order of expansion (${\mathrm{FSDTZ}}_{RS}^{\chi =1}$, ${\mathrm{EDZ}2}_{}^{\chi =1}$, and EDZ3). The first-order and second-order models are taken without the shear correction factor χ=1, as explained in [93,94,95]. The results of the displacement analysis are shown in Figure 20. In particular, both the tangential component $u_{1}^{\left(0\right)}$ (Figure 20a) and the vertical one $u_{3}^{\left(0\right)}$ (Figure 20b) increase when the damage affects wider areas, as expected. Similar graphs are obtained by the various HSDTs. The through-the-thickness variations of strains, stresses, and three-dimensional displacements are depicted respectively in Figure 21, Figure 22 and Figure 23, where the so-called zig-zag effect is clearly observable. In general, comparable profiles are obtained through all the structural models, even though the ${\mathrm{FSDTZ}}_{RS}^{\chi =1}$ provides curves that are slightly detached, especially when the plotted quantities present a lower order of magnitude.

### 8.5. Doubly-Curved Panel of Revolution

**Figure 24:**
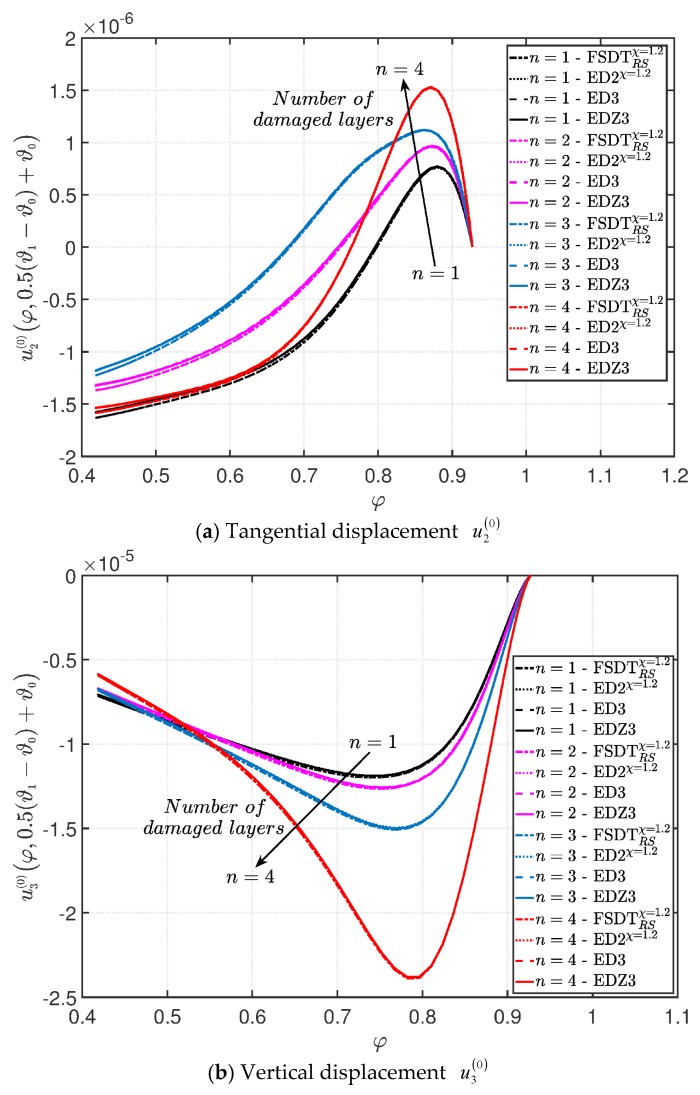
Evaluation of the central vertical displacement profile [m] for the CCCF laminated panel of revolution varying the mechanical parameters of the damage (Gau-6): (**a**) Tangential displacement $u_{2}^{\left(0\right)}$; (**b**) Vertical displacement $u_{3}^{\left(0\right)}$. The results are obtained by means of several HSDTs.

A branch of a parabolic arch is employed to describe the meridian curve of a CCCF doubly-curved panel of revolution (Figure 4c). As illustrated in the previous application, the meaning of the geometric parameters of the parabola listed in Table 3 is shown in Figure 4d for the sake of clarity. The damage is modeled using the mathematical expression denoted as Gau-6. In this circumstance, the origin of the Gaussian variation is located at $\alpha_{1m}^{\left(k\right)}=0.75\left(\varphi_{1}-\varphi_{0}\right)+\varphi_{0}$, $\alpha_{2m}^{\left(k\right)}=0.50\left(\vartheta_{1}-\vartheta_{0}\right)+\vartheta_{0}$, for k=1,2,…,n, where n represents the layers progressively involved by the damage (the count goes from the bottom to the upper layer of the laminated structure). Both the intensity and the size of the damage are kept constant in each ply. The displacement analysis is presented in Figure 24 for the tangential $u_{2}^{\left(0\right)}$ and vertical $u_{3}^{\left(0\right)}$ components, from which it is possible to observe that the plotted quantities, measured along the first coordinate φ for ϑ=0, increase by expanding the damage to the upper layers.

**Figure 25:**
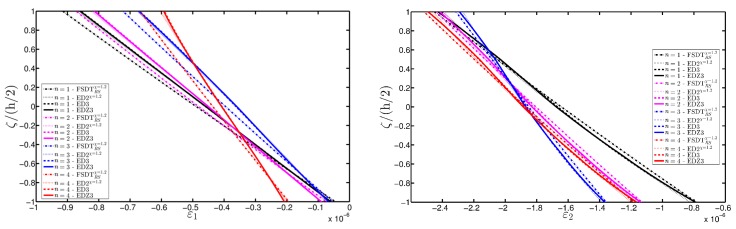

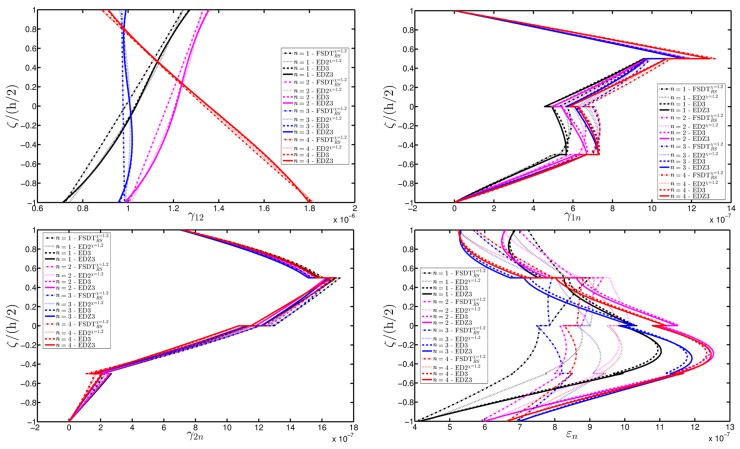
Through-the-thickness profiles of strain components for the CCCF doubly-curved panel of revolution computed at the point *C* for different HSDTs. The effect of the damage is investigated by varying the parameters of the function Gau-6.

**Figure 26:**
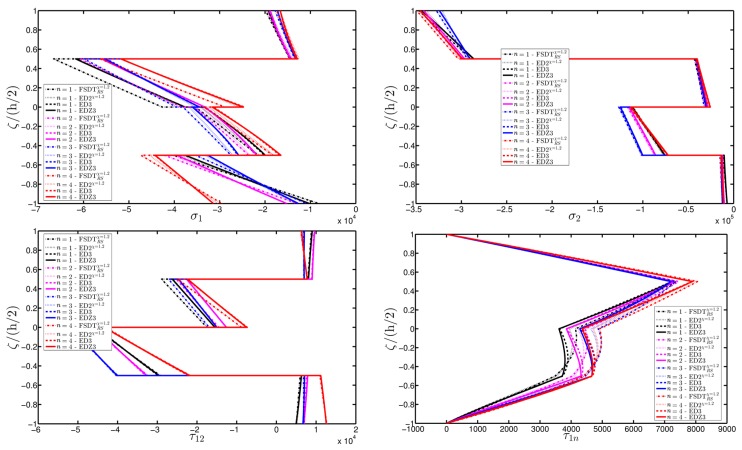

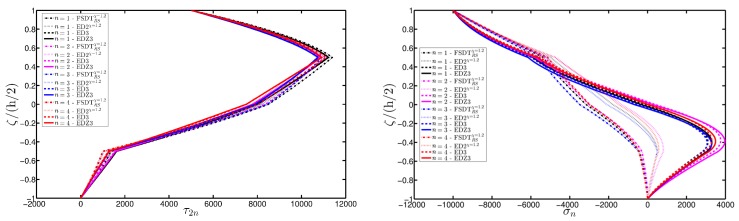
Through-the-thickness profiles of stress components [Pa] for the CCCF doubly-curved panel of revolution computed at the point *C* for different HSDTs. The effect of the damage is investigated by varying the parameters of the function Gau-6.

**Figure 27:**
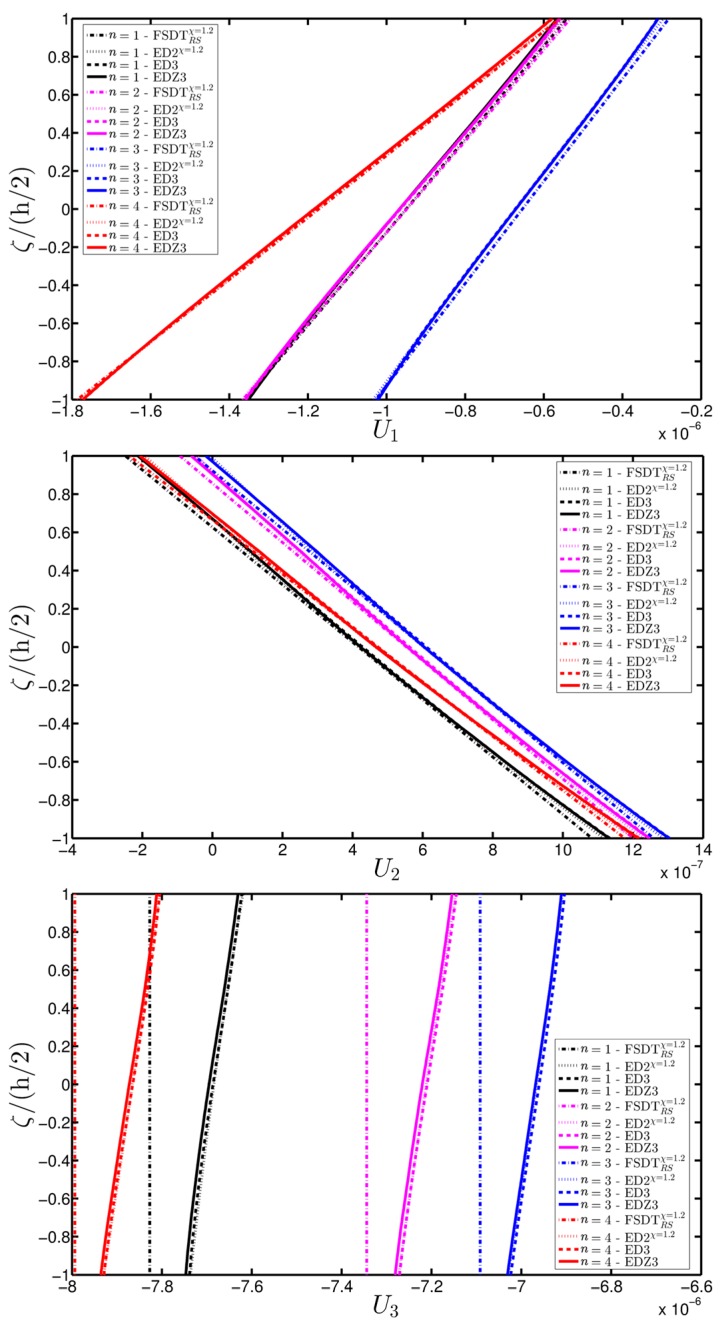
Through-the-thickness profiles of displacement components [m] for the CCCF doubly-curved panel of revolution computed at the point *C* for different HSDTs. The effect of the damage is investigated by varying the parameters of the function Gau-6.

The effect of the damage parameters on the strain, stress, and displacement profile along the shell thickness is investigated in Figure 25, Figure 26 and Figure 27. By comparing all the curves in these figures, it is possible to note that different through-the-thickness profiles are obtained varying the order of kinematic expansion of the structural theory. Such differences are more evident in the normal strains and stresses ($\varepsilon_{n}$ and $\sigma_{n}$, respectively). Finally, it should be noted that the boundary conditions at the external surface are always well-enforced for all the static analyses presented in this section.

## 9. Conclusions and Remarks

A numerical analysis was proposed in the paper to deal with the linear static behavior of damaged plates and shells. The solutions were obtained through the application of the GDQ method, which was also employed to evaluate the through-the-thickness variations of stress and strain components by means of a recovery procedure based on the three-dimensional equilibrium equations for a shell structure. Several boundary conditions, geometric shapes, stacking sequences, and load combinations were considered and investigated through a variety of higher-order structural models, including the Murakami’s function for the zig-zag effect when needed. The mechanical properties of the shells were taken as variable to model a damaged configuration. In particular, the two-dimensional Gaussian function and an elliptic expression were introduced to model a damage which affects all the engineering constants of the corresponding medium in concentrated areas of the shell domain. Several parametric investigations were carried out to study the effect of the various parameters that describe those functions. The following observations are noted:

- The results were presented in terms of displacement profiles related to the shell middle surface and through-the-thickness variations of strain, stress, and displacement components at specific points of the domain. It was proven that the damage affects all these static quantities.
- The numerical approach shows a convergent behavior when applied to this kind of structural problems.
- It was proven that the damage modifies the structural response. In general, the displacement increases when the damage intensity grows or when it involves a bigger area of the domain; analogously, the overall displacement is greater when the damage spreads to the adjacent layers.
- As far as stresses and strains are concerned, the effects of the damage on their through-the-thickness profiles are lower than the effects caused on the corresponding displacements; nevertheless, it is possible to observe noticeable differences between the profiles which are related respectively to the minimum and maximum values of the parameters used in the parametric analyses to model the deterioration of the mechanical properties.
- Similar profiles in terms of strains, stresses, and displacements are obtained by means of different HSDTs. Consequently, all these enriched kinematic models are able to deal with damaged composite plates and shells.
- No significant differences are observable between a higher-order model and the corresponding one embedded with the Murakami’s function when a laminated composite is analyzed. On the other hand, the Murakami’s function is required to deal with sandwich structures with an inner soft-core.

As a future development, the authors believe that the present damage model should be also employed in the dynamic field to investigate how the deterioration of the mechanical properties of a generic medium affects the corresponding structural response. In addition, the authors will attempt to compare the present approach with some experimental and numerical tests available in the literature.

## Figures and Tables

**Table 1:** Boundary conditions for the ESL model (τ=0,1,2,…,N,N+1).

Edge coordinates $\alpha_{2}^{0}\leq \alpha_{2}\leq \alpha_{2}^{1}$ and $\alpha_{1}=\alpha_{1}^{0}$ or $\alpha_{1}=\alpha_{1}^{1}$	Edge coordinates $\alpha_{1}^{0}\leq \alpha_{1}\leq \alpha_{1}^{1}$ and $\alpha_{2}=\alpha_{2}^{0}$ or $\alpha_{2}=\alpha_{2}^{1}$
**Clamped (C)**	
$u_{1}^{\left(\tau \right)}=u_{2}^{\left(\tau \right)}=u_{3}^{\left(\tau \right)}=0$	$u_{1}^{\left(\tau \right)}=u_{2}^{\left(\tau \right)}=u_{3}^{\left(\tau \right)}=0$
**Free (F)**	
$N_{1}^{\left(\tau \right)}=N_{12}^{\left(\tau \right)}=T_{1}^{\left(\tau \right)}=0$	$N_{21}^{\left(\tau \right)}=N_{2}^{\left(\tau \right)}=T_{2}^{\left(\tau \right)}=0$
**Simply-supported (S)**	
$N_{1}^{\left(\tau \right)}=0,\;u_{2}^{\left(\tau \right)}=u_{3}^{\left(\tau \right)}=0$	$u_{1}^{\left(\tau \right)}=0,\;N_{2}^{\left(\tau \right)}=0,\;u_{3}^{\left(\tau \right)}=0$

**Table 2:** Different kinds of discrete grid distributions for the Generalized Differential Quadrature (GDQ) method [130]. Symbols k and N stand for i and $I_{N}$ respectively, along the $\alpha_{1}$ direction. On the other hand, k and N specify j and $I_{M}$ respectively, along the $\alpha_{2}$ direction. Notations $L_{N},\;L_{N+1}$ are introduced to denote the corresponding Legendre polynomials.

**(a) Quadratic (Quad)**
$\left\{ \begin{array}{l} r_{k}=2{\left(\frac{k-1}{N-1}\right)}^{2},\;k=1,2,\ldots ,\frac{N+1}{2}\\ r_{k}=-2{\left(\frac{k-1}{N-1}\right)}^{2}+4\left(\frac{k-1}{N-1}\right)-1,\;k=\frac{N+1}{2}+1,\ldots ,N-1,N \end{array}\right.$
**(b) Chebyshev I kind (Cheb I)**
$r_{k}=\cos\left(\frac{2\left(N-k\right)+1}{2N}\pi \right),\;k=1,2,\ldots ,N,\;r\in \left[-1,1\right]$
**(c) Chebyshev II kind (Cheb II)**
$r_{k}=\cos\left(\frac{N-k+1}{N+1}\pi \right),\;k=1,2,\ldots ,N,\;r\in \left[-1,1\right]$
**(d) Legendre-Gauss (Leg-Gau)**
$r_{k}=\mathrm{roots}\;\mathrm{of}\;\left(1-r^{2}\right)\cdot L_{N-1}^{}\left(r\right),\;k=1,2,\ldots ,N,\;r\in \left[-1,1\right]$
**(e) Chebyshev-Gauss (Cheb-Gau)**
$\begin{array}{l} r_{\;1}=-1,\;r_{\;N}=1,\;r_{k}=\cos\left(\frac{2\left(N-k\right)-1}{2\left(N-2\right)}\pi \right),\\ \;k=2,3,\ldots ,N-1,\;r\in \left[-1,1\right] \end{array}$
**(f) Legendre-Gauss-Lobatto (Leg-Gau-Lob)**
$r_{k}=\mathrm{roots}\;\mathrm{of}\;\left(1-r^{2}\right)\cdot \frac{d}{dr}\left(L_{N}\left(r\right)\right),\;k=1,2,\ldots ,N,\;r\in \left[-1,1\right]$
**(g) Legendre (Leg)**
$\begin{array}{c} r_{k}=\mathrm{roots}\;\mathrm{of}\;L_{N+1}\left(r\right),\;k=1,2,\ldots ,N,\;r\in \left[-1,1\right]\\ \mathrm{with}\;L_{N+1}\left(r\right)=\frac{{\left(-1\right)}^{N}}{2^{N}N!}\frac{d^{N}}{dr^{N}}\left({\left(1-r^{2}\right)}^{N}\right) \end{array}$
**(h) Chebyshev-Gauss-Lobatto (Cheb-Gau-Lob)**
$r_{k}=\cos\left(\frac{N-k}{N-1}\pi \right),\;k=1,2,\ldots ,N,\;r\in \left[-1,1\right]$

**Table 3:** Geometric and mechanical properties of the structures.

**(a) Square Plate**
Position vector: $r\left(x,y\right)=x\;e_{\;1}+y\;e_{\;2}$, with $x\in \left[0,L_{x}\right]$, $y\in \left[0,L_{y}\right]$, $L_{x}=L_{y}=1\;m$
Boundary conditions: SSSS (for the convergence analyses) and CCCC (for the stress and strain recovery procedure)
Applied loads: $q_{3}^{\left(+\right)}=-10\;\mathrm{kPa}$
Thickness and lamination scheme (isotropic): h=0.05 m (Aluminum)
Thickness and lamination scheme (laminated): h=0.05 m, (−45/45), with $h_{1}=h_{2}=0.025\;m$ (Glass-epoxy)
Thickness and lamination scheme (laminated): h=0.05 m, (−45/0/45), with $h_{1}=h_{3}=0.015\;m$, $h_{2}=0.02\;m$ (Glass-epoxy)
Evaluation point for strains, stresses and displacements: $A\equiv \left(0.2569L_{x},\;0.2569L_{y}\right)$
**(b) Singly-Curved Cylindrical Panel with Parabolic Profile**
Position vector: $r\left(\varphi ,y\right)=\frac{k\tan\varphi }{2}\;e_{1}-y\;e_{2}+\frac{k\tan^{2}\varphi }{4}\;e_{3}$, with $\varphi \in \left[\varphi_{0},\varphi_{1}\right]$, $y\in \left[0,L_{y}\right]$, $\varphi_{0}=-0.78540$, $\varphi_{1}=0.78540$, $L_{y}=4\;m$, $k=\left(a^{2}-d^{2}\right)/b$, a=2 m, b=1 m, c=−2 m, d=0 m
Boundary conditions: FCFC
Applied loads: $q_{1}^{\left(+\right)}=10\;\mathrm{kPa}$, $q_{3}^{\left(+\right)}=-5\;\mathrm{kPa}$
Thickness and lamination scheme: h=0.16 m, (0/Foam/0), with $h_{1}=h_{3}=0.04\;m$ (Glass-epoxy), $h_{2}=0.08\;m$ (Foam)
Evaluation point for strains, stresses and displacements: $B\equiv \left(0.7431\left(\varphi_{1}-\varphi_{0}\right)+\varphi_{0},\;0.7431L_{y}\right)$
**(c) Doubly-Curved Panel of Revolution with Parabolic Meridian**
Position vector: $r\left(\varphi ,\vartheta \right)=\frac{k\tan\varphi \cos\vartheta }{2}\;e_{1}-\frac{k\tan\varphi \sin\vartheta }{2}\;e_{2}+\frac{k\tan^{2}\varphi }{4}\;e_{3}$, with $\varphi \in \left[\varphi_{0},\varphi_{1}\right]$, $\vartheta \in \left[\vartheta_{0},\vartheta_{1}\right]$, $\varphi_{0}=0.41822$, $\varphi_{1}=0.41822$, $\vartheta_{0}=-\pi /3$, $\vartheta_{1}=\pi /3$, $k=\left(a^{2}-d^{2}\right)/b$, a=3 m, b=2 m, c=1 m, d=0 m
Boundary conditions: CCCF
Applied loads: $q_{2}^{\left(+\right)}=5\;\mathrm{kPa}$, $q_{3}^{\left(+\right)}=-10\;\mathrm{kPa}$
Thickness and lamination scheme: h=0.16 m, (0/60/30/90), with $h_{1}=h_{2}=h_{3}=h_{4}=0.04\;m$ (Graphite-epoxy)
Evaluation point for strains, stresses and displacements: $C\equiv \left(0.2569\left(\varphi_{1}-\varphi_{0}\right)+\varphi_{0},\;0.7431\left(\vartheta_{1}-\vartheta_{0}\right)+\vartheta_{0}\right)$

**Table 4:** Mechanical properties of the materials.

**Aluminum**	**Foam**
Young’s modulus: E=70 GPa Poisson’s ratio: ν=0.3	Young’s modulus: E=0.232 GPa Poisson’s ratio: ν=0.2
**Glass-Epoxy**	**Graphite-Epoxy**
Young’s moduli: $E_{1}=53.78\;\mathrm{GPa}$, $E_{2}=E_{3}=17.93\;\mathrm{GPa}$ Shear moduli: $G_{12}=G_{13}=8.96\;\mathrm{GPa}$, $G_{23}=3.45\;\mathrm{GPa}$ Poisson’s ratios: $\nu_{12}=\nu_{13}=0.25$, $\nu_{23}=0.34$	Young’s moduli: $E_{1}=137.9\;\mathrm{GPa}$, $E_{2}=E_{3}=8.96\;\mathrm{GPa}$ Shear moduli: $G_{12}=G_{13}=7.1\;\mathrm{GPa}$, $G_{23}=6.21\;\mathrm{GPa}$ Poisson’s ratios: $\nu_{12}=\nu_{13}=0.3$, $\nu_{23}=0.49$

**Table 5:** Analytical expressions of the functions used to model the damage.

**(a) Function 1 (Gau-1)**
$\psi_{G}^{},\;\delta =0.99,\;\alpha_{1m}^{}=0.5\;m,\;\alpha_{2m}^{}=0.5\;m,\;\Delta_{1}^{}=\Delta_{2}^{}=\Delta =5,\;\rho_{12}^{}=0$
**(b) Function 2 (Gau-2)**
$\begin{array}{c} \psi_{G}^{},\;\delta ,\;\alpha_{1m}^{}=0.5\;m,\;\alpha_{2m}^{}=0.5\;m,\;\Delta_{1}^{}=\Delta_{2}^{}=\Delta =10,\;\rho_{12}^{}=0\\ \mathrm{Variation}:\;\delta =0.00,\;0.25,\;0.50,\;0.75,\;0.99 \end{array}$
**(c) Function 3 (Gau-3)**
$\begin{array}{c} \psi_{G}^{},\;\delta =0.75,\;\alpha_{1m}^{}=0.5\;m,\;\alpha_{2m}^{}=0.5\;m,\;\Delta_{1}^{}=\Delta_{2}^{}=\Delta ,\;\rho_{12}^{}=0\\ \mathrm{Variation}:\;\Delta =0,\;5,\;10,\;15,\;20 \end{array}$
**(d) Function 4 (Gau-4)**
$\begin{array}{c} \psi_{G}^{\left(k\right)},\;\delta^{\left(k\right)},\;\alpha_{1m}^{\left(k\right)}=0.5\;m,\;\alpha_{2m}^{\left(k\right)}=0.5\;m,\;\Delta_{1}^{\left(k\right)}=\Delta_{2}^{\left(k\right)}=\Delta^{\left(k\right)},\;\rho_{12}^{\left(k\right)}=0,\;\mathrm{for}\;k=1,2,3\\ \begin{array}{l} \mathrm{Case}\;(a)\;-\;\delta^{\left(k\right)}=0.00\\ \mathrm{Case}\;(b)\;-\;\delta^{\left(k\right)}=0.25,\;\Delta^{\left(k\right)}=5\\ \mathrm{Case}\;(c)\;-\;\delta^{\left(k\right)}=0.50,\;\Delta^{\left(k\right)}=10\\ \mathrm{Case}\;(d)\;-\;\delta^{\left(k\right)}=0.75,\;\Delta^{\left(k\right)}=15\\ \mathrm{Case}\;(e)\;-\;\delta^{\left(k\right)}=0.99,\;\Delta^{\left(k\right)}=20 \end{array} \end{array}$
**(e) Function 5 (Gau-5)**
$\begin{array}{c} \psi_{G}^{\left(k\right)},\;\delta^{\left(n\right)},\;\alpha_{1m}^{\left(k\right)}=0.5\;m,\;\alpha_{2m}^{\left(k\right)}=0.5\;m,\;\Delta_{1}^{\left(n\right)}=\Delta_{2}^{\left(n\right)}=\Delta^{\left(n\right)},\;\rho_{12}^{\left(k\right)}=0,\;\mathrm{for}\;k=1,2,3\\ \begin{array}{l} \mathrm{Case}\;(a)\;-\;\delta^{\left(n\right)}=0.00\;\mathrm{for}\;n=1,2,3\\ \mathrm{Case}\;(b)\;-\;\delta^{\left(n\right)}=0.99,\;\Delta^{\left(n\right)}=5\;\mathrm{for}\text{​ }n=1\\ \mathrm{Case}\;(c)\;-\;\delta^{\left(n\right)}=0.99,\;\Delta^{\left(1\right)}=10,\;\Delta^{\left(2\right)}=5,\;\mathrm{for}\;n=1,2\\ \mathrm{Case}\;(d)\;-\;\delta^{\left(n\right)}=0.99,\;\Delta^{\left(1\right)}=15,\;\Delta^{\left(2\right)}=10,\;\Delta^{\left(3\right)}=5\;\mathrm{for}\;n=1,2,3 \end{array} \end{array}$
**(f) Function 6 (Ell-1)**
$\begin{array}{c} \psi_{E}^{\left(k\right)},\;\delta^{\left(k\right)}=0.50,\;\alpha_{1m}^{\left(k\right)}=0.35\left(\varphi_{1}-\varphi_{0}\right)+\varphi_{0},\;\alpha_{2m}^{\left(k\right)}=2\;m,\;\Delta_{1}^{\left(k\right)},\;\Delta_{2}^{\left(k\right)}=1000,\;\rho_{12}^{\left(k\right)}=0,\;\mathrm{for}\;k=1,3\\ \begin{array}{l} \mathrm{Case}\;(a)\;-\;\Delta_{1}^{\left(k\right)}=0\\ \mathrm{Case}\;(b)\;-\;\Delta_{1}^{\left(k\right)}=10\\ \mathrm{Case}\;(c)\;-\;\Delta_{1}^{\left(k\right)}=30\\ \mathrm{Case}\;(d)\;-\;\Delta_{1}^{\left(k\right)}=50 \end{array} \end{array}$
**(g) Function 7 (Gau-6)**
$\begin{array}{l} \psi_{G}^{\left(k\right)},\;\delta^{\left(k\right)}=0.75,\;\alpha_{1m}^{\left(k\right)}=0.75\left(\varphi_{1}-\varphi_{0}\right)+\varphi_{0},\;\alpha_{2m}^{\left(k\right)}=0.50\left(\vartheta_{1}-\vartheta_{0}\right)+\vartheta_{0},\\ \;\Delta_{1}^{\left(k\right)}=\Delta_{2}^{\left(k\right)}=\Delta^{\left(k\right)}=20,\;\rho_{12}^{\left(k\right)}=0,\;\mathrm{for}\;k=1,2,\ldots ,n \end{array}$

**Table 6:** Convergence analysis in terms of central deflection [m] for the SSSS square plate increasing the number of grid points $I_{N}=I_{M}$ for several grid distributions, lamination schemes and Higher-order Shear Deformation Theories (HSDTs).

$I_{N}$	Cheb-Gau-Lob	Quad	Cheb I	Cheb II	Leg-Gau	Cheb-Gau	Leg-Gau-Lob	Leg
**Isotropic Plate (Aluminum)—**$FSDT_{RS}^{\chi =1.2}$								
21	−7.073 × 10^−5^	−1.154 × 10^−4^	−7.622 × 10^−5^	−1.353 × 10^−4^	−7.262 × 10^−5^	−7.545 × 10^−5^	−7.105 × 10^−5^	−9.173 × 10^−5^
23	−6.546 × 10^−5^	−9.973 × 10^−5^	−5.685 × 10^−5^	−3.301 × 10^−5^	−6.734 × 10^−5^	−6.909 × 10^−5^	−6.246 × 10^−5^	−4.732 × 10^−5^
25	−6.355 × 10^−5^	−8.846 × 10^−5^	−6.770 × 10^−5^	−1.003 × 10^−4^	−6.414 × 10^−5^	−6.515 × 10^−5^	−6.417 × 10^−5^	−7.731 × 10^−5^
27	−6.149 × 10^−5^	−8.023 × 10^−5^	−5.648 × 10^−5^	−3.504 × 10^−5^	−6.221 × 10^−5^	−6.282 × 10^−5^	−6.002 × 10^−5^	−4.902 × 10^−5^
29	−6.094 × 10^−5^	−7.432 × 10^−5^	−6.409 × 10^−5^	−8.665 × 10^−5^	−6.107 × 10^−5^	−6.141 × 10^−5^	−6.153 × 10^−5^	−7.095 × 10^−5^
31	−6.011 × 10^−5^	−7.006 × 10^−5^	−5.686 × 10^−5^	−3.779 × 10^−5^	−6.040 × 10^−5^	−6.061 × 10^−5^	−5.929 × 10^−5^	−5.086 × 10^−5^
33	−6.002 × 10^−5^	−6.703 × 10^−5^	−6.249 × 10^−5^	−8.078 × 10^−5^	−6.002 × 10^−5^	−6.012 × 10^−5^	−6.052 × 10^−5^	−6.794 × 10^−5^
35	−5.966 × 10^−5^	−6.487 × 10^−5^	−5.731 × 10^−5^	−4.035 × 10^−5^	−5.979 × 10^−5^	−5.986 × 10^−5^	−5.914 × 10^−5^	−5.231 × 10^−5^
37	−5.971 × 10^−5^	−6.335 × 10^−5^	−6.168 × 10^−5^	−7.783 × 10^−5^	−5.967 × 10^−5^	−5.970 × 10^−5^	−6.010 × 10^−5^	−6.631 × 10^−5^
39	−5.952 × 10^−5^	−6.227 × 10^−5^	−5.770 × 10^−5^	−4.244 × 10^−5^	−5.960 × 10^−5^	−5.962 × 10^−5^	−5.915 × 10^−5^	−5.341 × 10^−5^
41	−5.960 × 10^−5^	−6.150 × 10^−5^	−6.122 × 10^−5^	−7.589 × 10^−5^	−5.956 × 10^−5^	−5.957 × 10^−5^	−5.991 × 10^−5^	−6.525 × 10^−5^
43	−5.949 × 10^−5^	−6.096 × 10^−5^	−5.801 × 10^−5^	−4.414 × 10^−5^	−5.954 × 10^−5^	−5.954 × 10^−5^	−5.921 × 10^−5^	−5.426 × 10^−5^
45	−5.956 × 10^−5^	−6.058 × 10^−5^	−6.090 × 10^−5^	−7.430 × 10^−5^	−5.953 × 10^−5^	−5.953 × 10^−5^	−5.981 × 10^−5^	−6.444 × 10^−5^
47	−5.949 × 10^−5^	−6.030 × 10^−5^	−5.827 × 10^−5^	−4.556 × 10^−5^	−5.952 × 10^−5^	−5.952 × 10^−5^	−5.927 × 10^−5^	−5.496 × 10^−5^
49	−5.955 × 10^−5^	−6.010 × 10^−5^	−6.067 × 10^−5^	−7.290 × 10^−5^	−5.952 × 10^−5^	−5.952 × 10^−5^	−5.975 × 10^−5^	−6.380 × 10^−5^
51	−5.950 × 10^−5^	−5.996 × 10^−5^	−5.848 × 10^−5^	−4.678 × 10^−5^	−5.952 × 10^−5^	−5.952 × 10^−5^	−5.932 × 10^−5^	−5.554 × 10^−5^
**Laminated Plate (−45/45)—**$FSDT_{RS}^{\chi =1.2}$								
21	−1.662 × 10^−4^	−2.752 × 10^−4^	−1.759 × 10^−4^	−2.830 × 10^−4^	−1.709 × 10^−4^	−1.778 × 10^−4^	−1.661 × 10^−4^	−2.056 × 10^−4^
23	−1.532 × 10^−4^	−2.365 × 10^−4^	−1.343 × 10^−4^	−8.009 × 10^−5^	−1.577 × 10^−4^	−1.619 × 10^−4^	−1.465 × 10^−4^	−1.132 × 10^−4^
25	−1.481 × 10^−4^	−2.086 × 10^−4^	−1.561 × 10^−4^	−2.167 × 10^−4^	−1.496 × 10^−4^	−1.520 × 10^−4^	−1.491 × 10^−4^	−1.749 × 10^−4^
27	−1.430 × 10^−4^	−1.883 × 10^−4^	−1.322 × 10^−4^	−8.429 × 10^−5^	−1.447 × 10^−4^	−1.461 × 10^−4^	−1.398 × 10^−4^	−1.160 × 10^−4^
29	−1.414 × 10^−4^	−1.736 × 10^−4^	−1.476 × 10^−4^	−1.888 × 10^−4^	−1.418 × 10^−4^	−1.426 × 10^−4^	−1.425 × 10^−4^	−1.609 × 10^−4^
31	−1.394 × 10^−4^	−1.629 × 10^−4^	−1.325 × 10^−4^	−9.043 × 10^−5^	−1.400 × 10^−4^	−1.405 × 10^−4^	−1.376 × 10^−4^	−1.198 × 10^−4^
33	−1.390 × 10^−4^	−1.551 × 10^−4^	−1.438 × 10^−4^	−1.761 × 10^−4^	−1.390 × 10^−4^	−1.392 × 10^−4^	−1.399 × 10^−4^	−1.542 × 10^−4^
35	−1.381 × 10^−4^	−1.493 × 10^−4^	−1.333 × 10^−4^	−9.648 × 10^−5^	−1.384 × 10^−4^	−1.385 × 10^−4^	−1.370 × 10^−4^	−1.229 × 10^−4^
37	−1.381 × 10^−4^	−1.449 × 10^−4^	−1.419 × 10^−4^	−1.694 × 10^−4^	−1.380 × 10^−4^	−1.381 × 10^−4^	−1.388 × 10^−4^	−1.505 × 10^−4^
39	−1.376 × 10^−4^	−1.413 × 10^−4^	−1.340 × 10^−4^	−1.016 × 10^−4^	−1.378 × 10^−4^	−1.378 × 10^−4^	−1.369 × 10^−4^	−1.253 × 10^−4^
41	−1.377 × 10^−4^	−1.381 × 10^−4^	−1.408 × 10^−4^	−1.653 × 10^−4^	−1.376 × 10^−4^	−1.377 × 10^−4^	−1.383 × 10^−4^	−1.481 × 10^−4^
43	−1.375 × 10^−4^	−1.350 × 10^−4^	−1.346 × 10^−4^	−1.057 × 10^−4^	−1.376 × 10^−4^	−1.376 × 10^−4^	−1.369 × 10^−4^	−1.272 × 10^−4^
45	−1.376 × 10^−4^	−1.317 × 10^−4^	−1.401 × 10^−4^	−1.623 × 10^−4^	−1.375 × 10^−4^	−1.375 × 10^−4^	−1.380 × 10^−4^	−1.465 × 10^−4^
47	−1.374 × 10^−4^	−1.277 × 10^−4^	−1.351 × 10^−4^	−1.090 × 10^−4^	−1.375 × 10^−4^	−1.375 × 10^−4^	−1.370 × 10^−4^	−1.286 × 10^−4^
49	−1.375 × 10^−4^	−1.228 × 10^−4^	−1.395 × 10^−4^	−1.599 × 10^−4^	−1.374 × 10^−4^	−1.374 × 10^−4^	−1.378 × 10^−4^	−1.452 × 10^−4^
51	−1.374 × 10^−4^	−1.166 × 10^−4^	−1.354 × 10^−4^	−1.118 × 10^−4^	−1.374 × 10^−4^	−1.374 × 10^−4^	−1.370 × 10^−4^	−1.298 × 10^−4^
**Laminated Plate (−45/45)—**$FSDTZ_{RS}^{\chi =1.2}$								
21	−1.673 × 10^−4^	−2.773 × 10^−4^	−1.770 × 10^−4^	−2.829 × 10^−4^	−1.721 × 10^−4^	−1.791 × 10^−4^	−1.672 × 10^−4^	−2.064 × 10^−4^
23	−1.542 × 10^−4^	−2.383 × 10^−4^	−1.352 × 10^−4^	−8.037 × 10^−5^	−1.587 × 10^−4^	−1.629 × 10^−4^	−1.475 × 10^−4^	−1.140 × 10^−4^
25	−1.490 × 10^−4^	−2.103 × 10^−4^	−1.570 × 10^−4^	−2.168 × 10^−4^	−1.505 × 10^−4^	−1.530 × 10^−4^	−1.500 × 10^−4^	−1.756 × 10^−4^
27	−1.439 × 10^−4^	−1.898 × 10^−4^	−1.331 × 10^−4^	−8.469 × 10^−5^	−1.456 × 10^−4^	−1.470 × 10^−4^	−1.407 × 10^−4^	−1.168 × 10^−4^
29	−1.422 × 10^−4^	−1.749 × 10^−4^	−1.484 × 10^−4^	−1.890 × 10^−4^	−1.426 × 10^−4^	−1.434 × 10^−4^	−1.433 × 10^−4^	−1.617 × 10^−4^
31	−1.402 × 10^−4^	−1.641 × 10^−4^	−1.334 × 10^−4^	−9.092 × 10^−5^	−1.409 × 10^−4^	−1.413 × 10^−4^	−1.384 × 10^−4^	−1.205 × 10^−4^
33	−1.398 × 10^−4^	−1.561 × 10^−4^	−1.445 × 10^−4^	−1.763 × 10^−4^	−1.398 × 10^−4^	−1.400 × 10^−4^	−1.407 × 10^−4^	−1.548 × 10^−4^
35	−1.389 × 10^−4^	−1.500 × 10^−4^	−1.341 × 10^−4^	−9.702 × 10^−5^	−1.392 × 10^−4^	−1.393 × 10^−4^	−1.378 × 10^−4^	−1.237 × 10^−4^
37	−1.388 × 10^−4^	−1.453 × 10^−4^	−1.426 × 10^−4^	−1.698 × 10^−4^	−1.388 × 10^−4^	−1.388 × 10^−4^	−1.396 × 10^−4^	−1.511 × 10^−4^
39	−1.384 × 10^−4^	−1.412 × 10^−4^	−1.348 × 10^−4^	−1.022 × 10^−4^	−1.385 × 10^−4^	−1.386 × 10^−4^	−1.376 × 10^−4^	−1.261 × 10^−4^
41	−1.385 × 10^−4^	−1.374 × 10^−4^	−1.415 × 10^−4^	−1.657 × 10^−4^	−1.384 × 10^−4^	−1.384 × 10^−4^	−1.390 × 10^−4^	−1.488 × 10^−4^
43	−1.382 × 10^−4^	−1.335 × 10^−4^	−1.353 × 10^−4^	−1.063 × 10^−4^	−1.383 × 10^−4^	−1.383 × 10^−4^	−1.376 × 10^−4^	−1.279 × 10^−4^
45	−1.383 × 10^−4^	−1.290 × 10^−4^	−1.408 × 10^−4^	−1.628 × 10^−4^	−1.382 × 10^−4^	−1.382 × 10^−4^	−1.387 × 10^−4^	−1.472 × 10^−4^
47	−1.381 × 10^−4^	−1.235 × 10^−4^	−1.358 × 10^−4^	−1.096 × 10^−4^	−1.382 × 10^−4^	−1.382 × 10^−4^	−1.377 × 10^−4^	−1.293 × 10^−4^
49	−1.382 × 10^−4^	−1.165 × 10^−4^	−1.403 × 10^−4^	−1.604 × 10^−4^	−1.382 × 10^−4^	−1.381 × 10^−4^	−1.386 × 10^−4^	−1.459 × 10^−4^
51	−1.381 × 10^−4^	−1.076 × 10^−4^	−1.361 × 10^−4^	−1.124 × 10^−4^	−1.381 × 10^−4^	−1.381 × 10^−4^	−1.377 × 10^−4^	−1.305 × 10^−4^

## Appendix A

In order to facilitate the understanding of the presented higher-order model based on a variable kinematic expansion of the displacement field, in this section a complete list of the symbols and notations used in the paper are presented. In particular, the nomenclature employed in the previous sections is schematically presented in Table A1.

**Table A1:** Nomenclature.

Symbols	Definition
$R\left(\alpha_{1},\alpha_{2},\zeta \right)$	Three-dimensional position vector
$O^{\prime }\alpha_{1}\alpha_{2}\zeta$	Local reference system of origin $O^{\prime }$
$r\left(\alpha_{1},\alpha_{2}\right)$	Shell reference surface position vector
$n\left(\alpha_{1},\alpha_{2}\right)$	Outward unit normal vector
$R_{\;1},\;R_{\;2}$	Principal radii of curvatures
$A_{\;1},A_{\;2}$	Lamé parameters
$\alpha_{1},\alpha_{2}$	Principal curvilinear coordinates
h	Shell thickness
$U=U\left(\alpha_{1},\alpha_{2},\zeta \right)$	3D displacement component vector
$u^{\left(\tau \right)}=u^{\left(\tau \right)}\left(\alpha_{1},\alpha_{2}\right)$	τ-th order generalized displacement component vector
$F_{\tau }$	Thickness function
Z	Murakami’s function (zig-zag effect)
$\varepsilon^{\left(k\right)}=\varepsilon^{\left(k\right)}\left(\alpha_{1},\alpha_{2},\zeta \right)$	3D strain component vector
$D_{\Omega }$	Kinematic differential operator
$Z^{\left(\tau \right)}$	Matrix containing the terms related to the shell thickness
$\sigma^{\left(k\right)}=\sigma^{\left(k\right)}\left(\alpha_{1},\alpha_{2},\zeta \right)$	3D stress component vector
${\bar{C}}^{\left(k\right)}$	Constitutive operator
${\bar{E}}_{nm}^{\left(k\right)}$	Elastic constants of the stiffness matrix
$S^{\left(\tau \right)}=S^{\left(\tau \right)}\left(\alpha_{1},\alpha_{2}\right)$	τ-th order generalized stress resultant vector
$A^{\left(\tau \eta \right)}$	Stiffness matrix
κ	Shear correction factor
$D_{\Omega }^{*}$	Equilibrium differential operator
$L^{\left(\tau \eta \right)}$	Fundamental operator
$\Psi_{}^{\left(k\right)}$	Variation of the mechanical properties (damage)
$\psi_{G}^{\left(k\right)},\psi_{E}^{\left(k\right)}$	Gaussian and Elliptic variation (damage)
$\left(\alpha_{1m}^{\left(k\right)},\alpha_{2m}^{\left(k\right)}\right)$	Application point of the damage
$\Lambda_{1}^{\left(k\right)},\Lambda_{2}^{\left(k\right)}$	Size parameters of the damage
